# Comparative Analysis of Transposable Elements in Genus *Calliptamus* Grasshoppers Revealed That Satellite DNA Contributes to Genome Size Variation

**DOI:** 10.3390/insects12090837

**Published:** 2021-09-17

**Authors:** Muhammad Majid, Huang Yuan

**Affiliations:** College of Life Sciences, Shaanxi Normal University, Xi’an 710100, China; majidento07@snnu.edu.cn

**Keywords:** genus *Calliptamus*, repeatome analysis, satellitome evolution, repeat profiling

## Abstract

**Simple Summary:**

*Calliptamus* is a genus of grasshoppers belonging to the family Acrididae. The genus *Calliptamus* includes approximately 17 recognized species. *Calliptamus abbreviatus*, *Calliptamus italicus*, and *Calliptamus barbarus* are three species that are widely found in northern China. These species are polyphagous, feeding on a variety of wild plants as well as crops, particularly legumes. The genome sizes, phylogenetic position, and transcriptome analysis of the genus *Calliptamus* were already known previous to this research. The repeatome analysis of these species was missing, which is directly linked to the larger genome sizes of the grasshoppers. Here, we classified repetitive DNA sequences at the level of superfamilies and sub-families, and found that LINE, TcMar-Tc1 and Ty3-gypsy LTR retrotransposons dominated the repeatomes of all genomes, accounting for 16–34% of the total genomes of these species. Satellite DNA dynamic evolutionary changes in all three genomes played a role in genome size evolution. This study would be a valuable source for future genome assemblies.

**Abstract:**

Transposable elements (TEs) play a significant role in both eukaryotes and prokaryotes genome size evolution, structural changes, duplication, and functional variabilities. However, the large number of different repetitive DNA has hindered the process of assembling reference genomes, and the genus level TEs diversification of the grasshopper massive genomes is still under investigation. The genus *Calliptamus* diverged from *Peripolus* around 17 mya and its species divergence dated back about 8.5 mya, but their genome size shows rather large differences. Here, we used low-coverage Illumina unassembled short reads to investigate the effects of evolutionary dynamics of satDNAs and TEs on genome size variations. The Repeatexplorer2 analysis with 0.5X data resulted in 52%, 56%, and 55% as repetitive elements in the genomes of *Calliptamus barbarus*, *Calliptamus italicus*, and *Calliptamus abbreviatus*, respectively. The LINE and Ty3-gypsy LTR retrotransposons and TcMar-Tc1 dominated the repeatomes of all genomes, accounting for 16–35% of the total genomes of these species. Comparative analysis unveiled that most of the transposable elements (TEs) except satDNAs were highly conserved across three genomes in the genus *Calliptamus* grasshoppers. Out of a total of 20 satDNA families, 17 satDNA families were commonly shared with minor variations in abundance and divergence between three genomes, and 3 were *Calliptamus barbarus* specific. Our findings suggest that there is a significant amplification or contraction of satDNAs at genus phylogeny which is the main cause that made genome size different.

## 1. Introduction

Eukaryotic genomes are composed of a large number of different repetitive DNA sequences [1,2,3]. Based on their distribution and arrangements among genomes, these are classified into two important groups: tandem repeats and interspersed repeats [4]. Tandemly repeated non-coding DNAs of TEs are a very dynamically fast-evolving part of genomes [5]. To describe the whole collection of different satDNA families in a genome, the term “satellitome” was proposed [6]. SatDNA has been further classified into minisatellites, microsatellites, and satellites on the basis of nucleotide sequences [4,7]. Other than the tandem repeats, transposable elements have been categorized into two main classes according to their mode of transposition. Class I retrotransposons multiply in a “copy and paste” fashion using the reverse transcriptase enzyme from RNA intermediate back into DNA [8]. Class II elements are well-studied groups of repeats; their mode of transposition is “cut and paste”, requiring no RNA intermediary; thus, they are referred to as transposons. Transposons are classified into several superfamilies according to their similarities [9,10].

One of the TEs group LTR retrotransposons is categorized into two superfamilies, Ty3_gypsy and Ty1_copia. Due to their high diversity of nucleotide sequences, these are further divided into a vast number of families which are generally single or groups of closely related species [11]. Generally, non-LTR are more frequently present in mammals and LTR are more abundant in plants [12,13].

The variation in genome size among species might reflect the contribution of various evolutionary strategies [14]. Transposable elements are the most important component of higher plant genomes, ranging from 50–90% [15]. The human genome contains 50% [7] retrotransposons, transposons, and satellite repeats considered as primary factors for genome size variation. This kind of rapid and concerted evolution gives rise to genomes or species-specific sequences [16]. Transposable elements were once recognized as non-functional and junk DNA sequences and are now considered as the main component of genome evolutions [17]. Multiple processes are involved in genome evolution, including polyploidy, whole genome duplications, restructuring chromosomes through inversion, translocation, fusion and fission, and complete loss or amplification of repetitive sequences and genes causing DNA mutations [18,19].

SatDNAs in insects have been identified in a very limited number of species by using the conventional method [20]. In the past, poor and limited descriptions of repeatomes and satellitomes were available. Now, repetitive DNA study has improved significantly since the introduction of next-generation sequencing technologies [21]. Investigation of highly complex populations of repetitive DNA sequences in plants has become possible due to the power of next-generation sequencing technologies (NGS), which can generate gigabases of data in a single run [22,23,24,25]. Next-generation sequencing technologies boost the process of genome assemblies, such as Illumina sequencing [26]. Complete characterization of satellitome and TEs composition in genomes have previously not been characterized in many species of insects. In grasshoppers, the presence of satDNAs and multi-gene families 5S and 45S rDNA, and H3-H4 histone genes have been reported in *Locusta migratoria* [6,27], *Eyprepocnemis plorans* [28], *Abracris flavolineata* [29], *Dichroplus pratensis* [30], *Rhammatocerus brasiliensis* [31,32], *genus Isophya* [33], and in the Coleoptera order *Tribolium castaneum* [34].

Availability of next-generation sequencing data allows extraordinary opportunities to access, detect and quantify the repetitive DNA sequences in genomes [25]. Recent work done by Ruiz-Ruano et al. [6] is a good example of in-depth high throughput analysis of satellitomes in the grasshopper genome using the RepeatExplorer pipeline. RepeatExplorer is a graph-based clustering of NGS data, which is very useful in the identification and classification of different types of repetitive elements [35,36]. This platform has already been applied to various plant genomes, including onion, camellia, cucumber, and potatoes [37,38,39]. However, de novo whole genome shotgun techniques are largely incapable of recovering highly repetitive regions such as centromeres and pericentromeric regions in their entirety, and, as a consequence, satDNAs are frequently mischaracterized or lack such assemblies [40]. RepeatExplorer2 performs similarity-based clustering on raw short sequencing reads and partial consensus assembly, enabling the identification of repeats even with sparse genome coverage samples. RepeatExplorer2 latest advancements include the TAREAN platform for identifying tandem repeats specifically by scanning for circular structures in directed read clusters.

*Calliptamus* is a genus of grasshoppers belonging to the family Acrididae. The genus *Calliptamus* includes approximately 17 recognized species. *Calliptamus abbreviatus*, *Calliptamus italicus*, and *Calliptamus barbarus* are three species that are widely found in northern China. These species are polyphagous by nature, feeding on a variety of wild plants as well as crops, particularly legumes. The sporadic infestations have been observed on cereals and grapevines. The genus *Calliptamus* separated from *Peripolus* approximately 17 mya, and its species divergence occurred at approximately 8.5 mya [41]. In light of this recent divergence and their distinct genome sizes with *Calliptamus barbarus* (10.37 pg), *Calliptamus italicus* (10.1 pg), and *Calliptamus abbreviatus* (9.99 pg) [42], we decided to work on this genus with the primary purpose of determining why congeneric species have varying genome sizes and what causes these discrepancies. Given the short time interval between subsequent species-species divergence, we anticipated that some satellite DNA evolution would occur between the three species. In the present comparative study, we used low-coverage Illumina unassembled short reads to investigate the evolutionary dynamics of satDNAs and TEs using RepeatExplorer2.

## 2. Materials and Methods

### 2.1. Sample Collection, Genome Size Estimation, and Next-Generation Sequencing

*Calliptamus* grasshopper samples were collected from different areas of China (Table S1). The samples were stored at −80 degrees Celsius to keep them usable for further DNA extraction and genomic analysis at the laboratory of molecular evolutionary biology, College of Life Science, Shaanxi Normal University. Freshly collected samples were used to estimate the genome size using flow cytometry (FCM) of propidium iodide-stained nuclei following the standard protocol. After the genome size estimation, the samples were sequenced using Illumina sequencing with a 350 bp insertion library and PE-150. Details are provided in Supplementary Materials (Table S1).

### 2.2. Quality Check, Random Sampling, and Pre-Processing of the Sequenced Data

The pair-end reads of 150 bp that are generated by WGS (Whole Genome Shotgun) sequencing were used in RepeatExplorer2 analysis. Genome coverage recommended for analysis is 0.01–0.5x [36,40]. We performed random sampling using the SeqTK tool (https://github.com/lh3/seqtk, accessed on 22 June 2020), making sure the sample truly represents the whole genome and extracted 10 million reads for repeat analysis from each sample. Data was uploaded to the Repeatexplorer2 galaxy server using the FTP upload option. The quality of the data was checked using the FastQC tool implemented in the RepeatExplorer2 galaxy instance. Pre-processing of fastq files was done by the “preprocessing of fastq paired reads” tool using the default setting implemented in the RepeatExplorer galaxy platform. Pre-processing includes trimming, quality filtering of the reads, discarding the single reads and keeping complete pairs, cut-adapt filtering, and interlacing of two fastq files.

### 2.3. Species Code Assignment, Reads Sub-Sampling, Concatenation and Comparative RepeatExplorer2 and TAREAN Clustering Analysis

The comparative analysis was performed following the protocol described by Novak et al. [43]. We used the RepeatExplorer2 utility tool “FASTA read name affixer” to assign three-letter species-specific prefixes to the read names. To execute this step, the first three capitalized letters of each species name were used as a prefix (*Calliptamus italicus* = “ITA”, *Calliptamus barbarus* = “BAR”, and *Calliptamus abbreviatus* = “ABR”), with the other settings set as default. Next, we used RepeatExplorer Utilities’ “Read Samples” tool to further perform sub-sampling. After setting the standard parameters, we selected the interleaved FASTA files and set the "number of reads" of each file to 500,000 and set the random seed number to 10. The concatenation of all three species datasets was performed using the “Text manipulation-concatenate datasets” tool. We gradually selected and inserted all three species files with the coded reads in the order of ITA, BAR, ABR and executed the tool. The concatenated FASTA file was created with 1.5 million reads. 

The concatenated FASTA file created in the previous step was used as input data for the RepeatExplorer2 and TAREAN (tandem repeat analyzer) tools (http://repeatexplorer.org/?page_id=818, online accessed on 2 July 2020) for clustering analysis. For the comparative mode of RepeatExplorer2 clustering, we set the parameters as, pair-end reads = yes, sample size = 1.5 million reads, reference database = Metazoa version 3.0, select queue = “long”, in advance options “comparative analysis = YES”, custom database “Repbase”, and group code length = ‘3’. For the tandem repeat analyzer (TAREAN), we used the default settings, sample size = 1.5 m reads and select queue = basic and fast. Three files resulted as an output of both clustering analysis: a log file, an HTML report, and an HTML archive report. HTML archive reports were downloaded for further inspection. The comparative visualization of Repeatexplorer2 transposable elements (TEs) results was created with “plot_comparative_clustering_summary.R” script using two output files from the RepeatExplorer pipeline (https://github.com/kavonrtep/revis, accessed on 7 July 2020).

### 2.4. Homology Searches, Comparative Satellitome Analysis, and Z-Score Values

Repeatexplorer2 results were manually inspected and unclassified clusters with spherical or ring-like graphs subjected to the YASS tool (https://bioinfo.lifl.fr/yass/index.php, accessed on 7 August 2020) to search for tandem repeats [44]. Likewise, based on homology, we tried to classify satellite DNAs into subfamilies by doing all to all comparisons using the ‘rm_homology.py’ script from the satminer toolkit (https://github.com/fjruizruano/satminer, accessed on 8 August 2020). As a result, each of the satDNAs families was given a name based on the nomenclature proposed by Ruiz et al. We skipped the species name because most of the satellite DNA families were commonly shared and the genus *Calliptamus* (C) first letter followed the word “Sat” and a number in order of decreasing genome proportion, followed by the consensus monomer length, e.g., CSat01-880. Using the Censor tool (http://www.girinst.org/, accessed on 8 August 2020), we searched for homology to each satellite DNA in the already-existing TEs Repbase database. Firstly, we checked for homology by selecting the arthropods section from the Repbase database. Then we searched all the databases for any similarity to consensus sequences of SatDNAs. We also searched for any similarity or coding sequences contained in each satellite DNA family against Dfam database and in NCBI/GenBank DNA databases using the BLAST tool.

We used RepeatMasker (http://repeatmasker.org, accessed on 11 September 2020) with the “-a” option and the RMBlast search engine to estimate the divergence and abundance of each satDNA. We selected 2 million reads at random and aligned them against the entire set of satDNA consensus sequences using the customized reference library (-lib) option. We calculated the average divergence for each species using the “calcDivergenceFromAlign.pl” script and created a satellitome landscape by using the “createRepeatLandscape.pl” from the RepeatMasker suite. To assess the overall gain and loss of each satDNA, we calculated the standardized Z-score values of abundance and divergence in an Excel spreadsheet.

### 2.5. Comparative Repeat Profiling of Satellite DNA

We used the RepeatProfiler tool (https://github.com/johnssproul/RepeatProfiler, accessed on 18 January 2021) for creating, visualizing, and comparing repetitive DNA profiles of each satDNA and rDNA from low-coverage short-read sequence data of three *Calliptamus* species. For this repeat profile analysis, satDNAs FASTA files were used as a reference sequence to map against the randomly selected 5 million reads from each sample. As we were comparing each satDNA profile across three species, we used the correlation analysis feature to compare the profiles against different samples. To assign our samples a group name, we used the “pre-corr” flag to auto-generate an input file (user_groups.txt) which was essential for the correlation analysis, and kept all other settings as defaults [45].

## 3. Results

### 3.1. Individual Clustering Analysis and Composition of Repeat among Three Genomes

**Figure 1:**
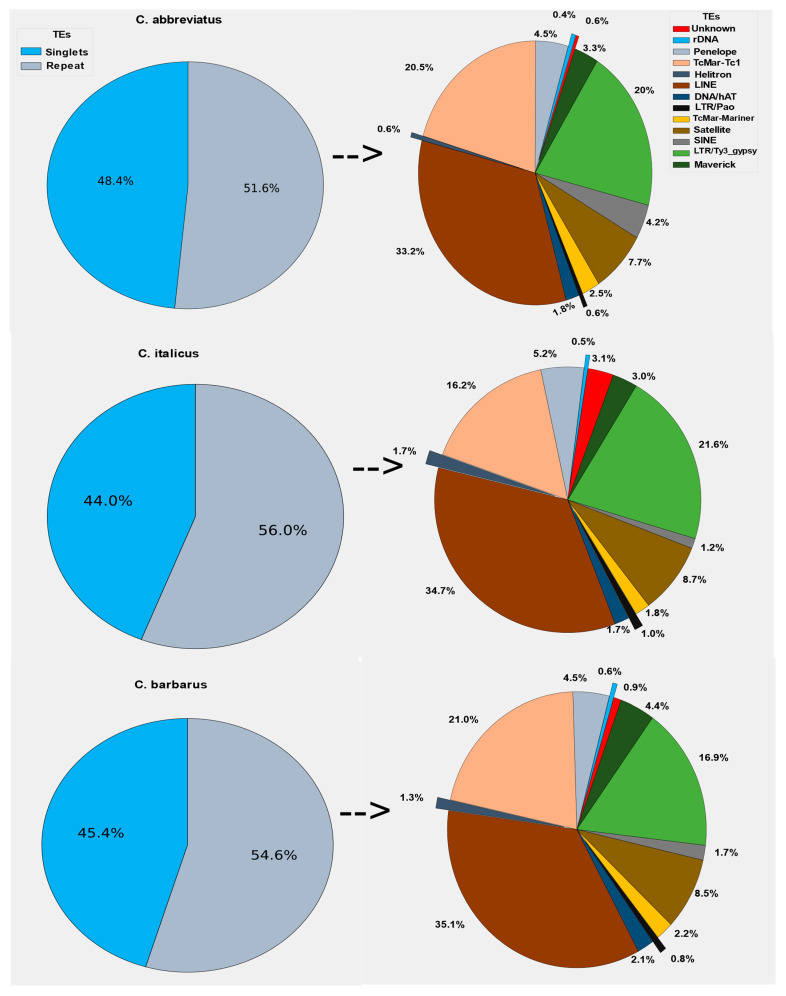
Genome composition of repetitive DNA sequences and singlets or unique sequences of three *Calliptamus* species. The genome repetitive part was dominated by LINE, LTR/Ty3_gypsy, and TcMar-Tc1 families.

In individual clustering analyses, the RepeatExplorer2 pipeline was used with the maximum recommended genome coverage of 0.5x per sample. On average, the majority of reads for *Calliptamus abbreviatus*, *Calliptamus italicus*, and *Calliptamus barbarus* were grouped into 31,533, 63,326, 58,181 clusters, and 31,466, 63,297, 58,146 Superclusters, respectively. Similarly, corresponding to 52%, 56%, and 55% of the genomes were repetitive elements, while singlets represented the remaining 48%, 44%, and 45% of the genomes, respectively (Supplementary Materials Figure S1). The LINE, Ty3-gypsy LTR retrotransposons, and DNA repeat TcMar-Tc1 dominated the repeatomes of all genomes, accounting for 16–35% of the total repetitive part of the genomes (Figure 1).

In the *Calliptamus italicus*, *Calliptamus abbreviatus*, and *Calliptamus barbarus* genomes, the most abundant TE element was LINE, which accounted for nearly 34.7%, 33.2%, and 35.1% of the total repetitive component, respectively. Ty3-gypsy, satellitome, and Penelope occupied 21.1%, 8.7%, and 5.2% of the whole repetitive share of the genome in *Calliptamus italicus*, which is collectively almost similar to that of *Calliptamus barbarus*. Other elements, such as SINE, Helitron, rDNA, Bel-pao, and Maverick, shared a very small fraction of the repetitive part (1.5% to 5%). The maximum number of reads is reported in the first hundred clusters, and they have a higher rate of annotation. There is a direct relationship between the number of reads and the number of annotations, because the annotations of clusters decrease as the number of reads in the cluster decreases, and these clusters are reported as unclassified (Figure S1).

### 3.2. Comparative Visualization of Repeat Content in Genus Calliptamus Species

**Figure 2:**
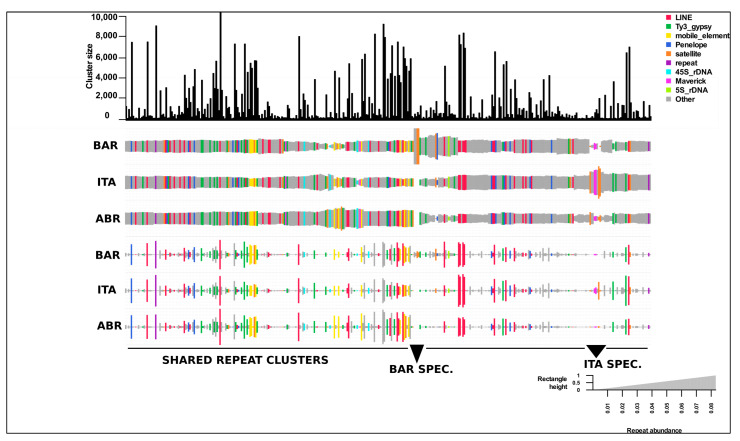
A comparative repeat graph of genus *Calliptamus* species. A bar plot on the top of the graph depicts the sizes (numbers of reads) of individual top clusters. The size of the rectangle is equal to the number of reads in a cluster for each species. Hierarchical clustering was used to sort the Clusters and species. The final annotation of the clusters is used to color unique rectangles. Species abrreviated with codes as *C. barbarus*; BAR, *C. italicus*; ITA, *C. abbreviatus*; ABR.

The total number of reads in the top cluster reflects a specific family of repeating elements, as shown in the top bar graph (see Figure 2). The proportions of reads from different species in the cluster were displayed as scaled rectangles below the bar graph. Due to the short time scale of divergence between these species, most of the top clusters reflect similar intensity of repetitions and are commonly shared among all three species, as expected. The clusters labeled as “shared” below the graph comprise the LTR-retrotransposon lineage, LINE, Ty3-gypsy, Penelope, and other mobile elements. A small part of the cluster contains repeats unique to each species, classified as “ITA-SPEC” and “BAR-SPEC”, mainly satellite repeats, which are the most dynamic parts of repeated DNA in evolution (Figure 2). When we manually inspected the unique regions of *Calliptamus barbarus* in the graph, we discovered four different clusters, two of which were satellite DNA repeats and the other two were unclassified. In addition, one of the unclassified clusters showed a ring-like graphical structure and dimer sequences of this cluster were subjected to the YASS tool to find the tandem repeats. The presence of the diagonal lines confirmed the satellite DNA family (Figure S3). The *Italicus* specific region included Maverick, unclassified, and satellite repeats. As most of the LINE and Ty3-gypsy clusters in this genus are relatively large, diversification and amplification of these families may result in larger genome sizes compared to other closely related genera (Figure 2). The genome sizes of *Calliptamus barbarus*, *Calliptamus italicus*, and *Calliptamus abbreviatus* determined by flow cytometry were 10.37, 10.1, and 9.99 pg, respectively (see Supplementary Table S1).

#### 3.2.1. Satellite DNA Characterization and Homology Searches

The Repeatexplorer2 and TAREAN tools implemented on the Galaxy platform discovered 20 satellite DNAs. We tried to classify satellite DNA based on homology, and grouped them into different superfamilies, but did not find the satellite DNA similarity greater than 50%.

#### 3.2.2. The Estimation of satDNA Abundance, Divergence, and Copy Number

In comparative satellitome analysis, the size of the satellite DNA family ranges from the smallest 26 nt CSat06-26 family to the largest satellite DNA family CSat13-2150 (2150 nt) recorded in Orthoptera. The A+T content of the satellite DNA family ranges from 39% to 61%, with a median value of 53.5%. The G+C content of two satellite DNA families, CSat12-42 and CSat13-2150, was estimated to be 61% and 51%, respectively (Table 1). All others were rich in A+T content and we did not observe any significant correlation between monomer repeat unit length and A+T content (Spearman rank-order correlation test: rs = −0.322, t = 1.40, *p* = 0.17, Supplementary Materials Figure S4).

The abundances of satellite DNA families in *Calliptamus italicus, Calliptamus barbarus* and *Calliptamus abbreviatus* ranged from 0.00008–0.31%, 0.00008–0.32% and 0.006–0.47%, respectively. Similarly, the total share of satDNAs accumulated in the genomes was 1.59, 1.59, and 2.07%, respectively. CSat01-800, CSat02-138, CSat03-159 and CSat09-130 satellite DNA families had a higher contribution to satellite DNA content in *Calliptamus italicus*. The *Calliptamus barbarus* satellite DNA abundance was dominated by the CSat01-800, CSat02-138, and CSat09-130 families. Similarly, the CSat01-800, CSat02-138, CSat09-130, and CSat10-1246 families accounted for half of the total satellite abundance of *Calliptamus abbreviatus* species. There was no significant correlation of abundance observed against monomer repeat unit length (*Calliptamus italicus*: rs = 0.21, t = 0.92, *p* = 0.36, *Calliptamus barbarus*: rs = −0.233, t = 0.336, *p* = 0.31, and *Calliptamus abbreviatus*: rs = −0.311, t = 0.196, *p* = 0.44).

On average, the K2P genetic divergence between satDNA families in *Calliptamus italicus* was 8.56%, 9.26% in *Calliptamus barbarus*, and 9.17% in *Calliptamus abbreviatus*. The most divergent satDNA family in *Calliptamus italicus* was CSat06-26 (18.61%), CSat08-294 with K2P (19.21%) in *Calliptamus barbarus*, and CSat06-26 with K2P (20.11%) in *Calliptamus abbreviatus* (see Table 1). There was no significant correlation observed between K2P divergence against monomer length (rs = −0.267, t = 0.270, *p =* 0.26) and A+T content (rs = 0.02, t = 0.09, *p =* 0.92) in *Calliptamus italicus* species. Unlike *Calliptamus italicus*, the K2P divergence has shown a positive correlation with monomer length (rs = 0.477, t = 0.03, *p =* 0.03) in *Calliptamus barbarus* and in *Calliptamus abbreviatus* (rs = 0.531, t = 0.023, *p =* 0.023) (Supplementary Information Figure S4).

#### 3.2.3. Comparison of Satellitome Landscapes

**Figure 3:**
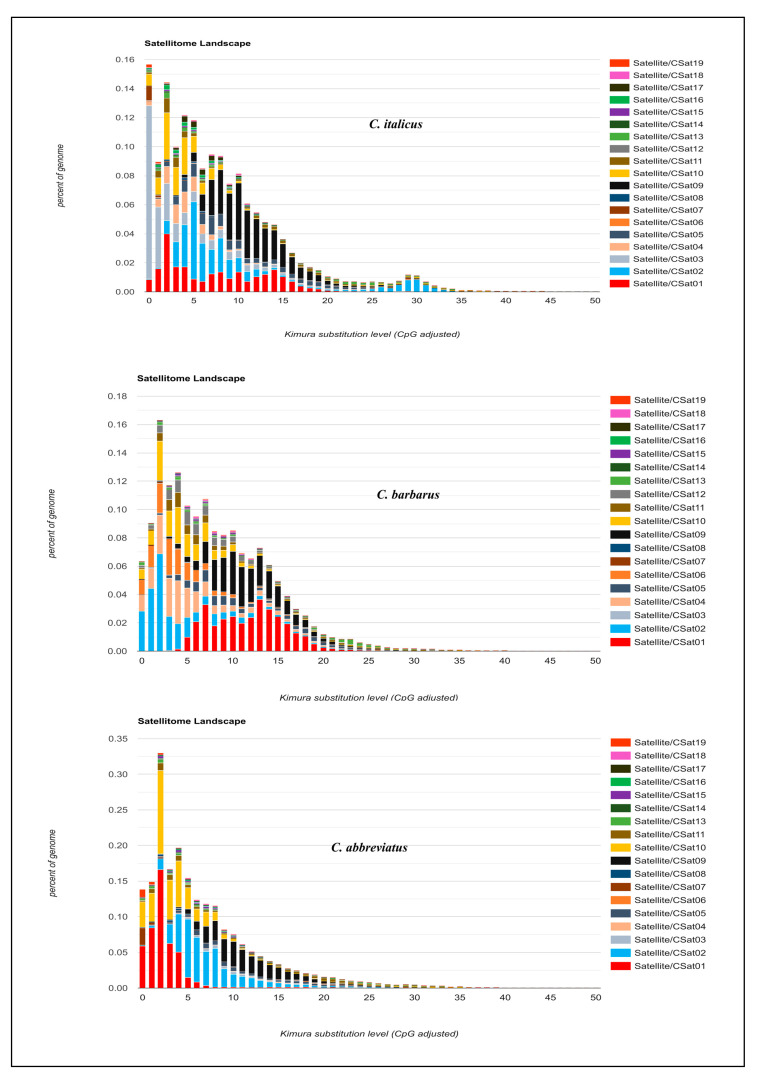
Satellitome landscape graphs represent genome coverage/genome proportion for each satDNA family along the *Y*-axis in the different genomes analyzed and Kimura genetic distances to their corresponding consensus sequence along the *X*-axis (K2P ranged from 0–50%).

Individual satellitome landscapes of the satDNA families for each of three species are shown in Figure 3. The copies clustered on the left of the graph deviate little from the consensus sequence, suggesting recent copies, while the sequences on the right represent old or degenerated copies. The peak of the graphs in all three satellitome landscapes was observed at 5% K2P genetic divergence, implying that most satellite DNA families have not diverged from the consensus sequences and the homogenization process is underway. This comparison of satellite DNA families among three species reveals two key points (Figure 3). First, the individual comparison reconfirmed the Repeatexplorer2 results of species specificity of each satellite DNA between different species. Secondly, the evaluation of monomeric variation showed double peak patterns for some families, indicating the presence of two different repeating units with dissimilar divergence rates.

**Figure 4:**
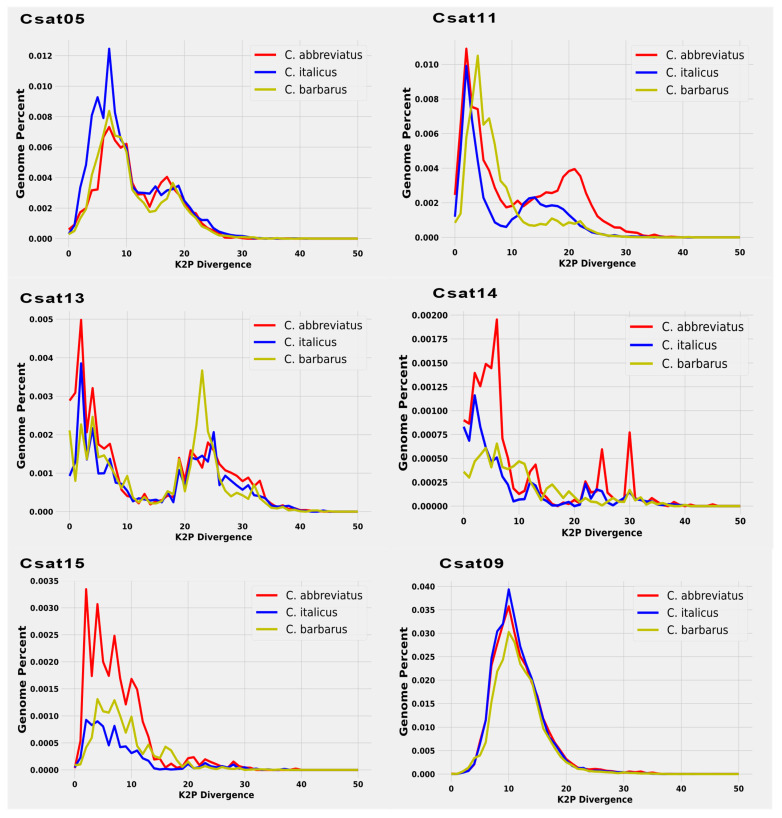
A comparative repeat line graph for each satDNA family revealed a two-peak pattern of divergent monomer sequences from consensus sequences across three species genomes. A satellite DNA family was highly conserved among the three species of the genus *Calliptamus* (CSat09).

Consistently, CSat05-270 repeat showed two peaks in *Calliptamus italicus* and *Calliptamus barbarus*, one pointing to very low divergent sequences, and the other approximately at 15% divergence. Similarly, CSat12-42 and CSat13-2150, and CSat11-220 except in *C. barbarous* showed two types of abundant repeats differing in divergence, reflecting that they may have different periods or homogenization tendencies. A CSat09-130 family with a divergent peak at 10% was found to be a highly conserved family across three species genomes with similar divergence and abundance (Figure 4).

Compared with the other two species, the CSat14-862 and CSat15-1533 families showed more than two peaks in *Calliptamus abbreviatus*, indicating the presence of more than two strongly diverging repeating units. CSat02-138, CSat10-1246, CSat16-225, and CSat18-185 only showed one less divergent peak among all the species with small variation in abundance. CSat03-159 exhibited a single peak below 5% in *Calliptamus italicus*, a flat distribution in *Calliptamus abbreviatus,* and was completely absent in *Calliptamus barbarus*. The *Calliptamus barbarus* specific satDNA families CSat06-26 and CSat12-42 also showed a single peak at 5% divergence. The abundantly different CSat04-181 family depicted a single peak in *Calliptamus italicus* and *Calliptamus barbarous* (Figure S5). A supporting document contains additional details on individual satDNA family RepeatExplorer2 clustering graphs (Figure S6).

### 3.3. Z-Score Abundance and Divergence and Repeat Profiling of Satellite DNAs/rDNAs

**Figure 5:**
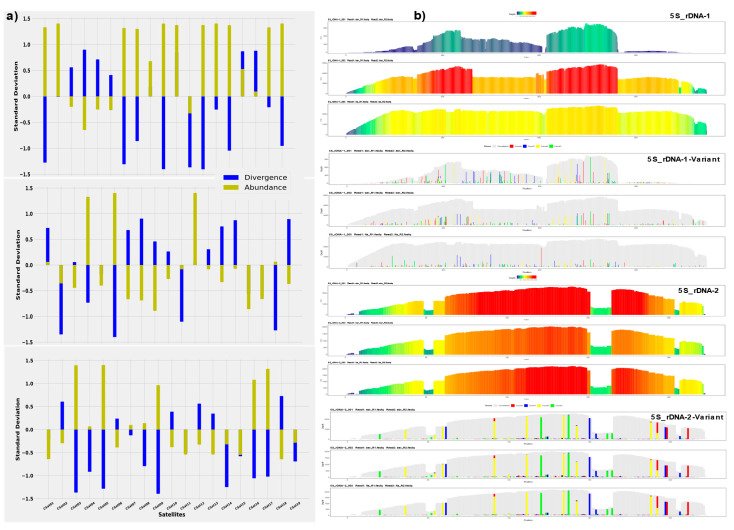
The standardized Z-score divergence and abundance values of each satDNA show the overall gain and loss (**a**) and the color-enhanced profiles of the 5S-rDNA across the three genomes. A 12 bp valley in the middle of the monomer site of (153–164) in the 5S-rDNA-2 profiles. Species profiles are arranged in a sequence of *Calliptamus abbreviatus => Calliptamus barbarus => Calliptamus italicus* (**b**).

The assessment of standardized Z-score abundance and divergence values of each satDNAs showed some fascinating evolutionary dynamics among these species. We estimate that the combination of positive Z-abundance and negative Z-divergence values indicates recent amplification of each satDNA. Conversely, the combination of negative Z-abundance and positive Z-divergence implies that the increase in divergence is caused by point mutation. The Z-score divergence and abundance values revealed the amplification of CSat01-800 and CSat02-138 in only *Calliptamus abbreviatus* species, and contraction for *Italicus and Barbarus*. CSat04-181 and CSat05-270 showed amplification (homogenization) only for *Calliptamus barbarus* and *Calliptamus italicus*, respectively (Figure 5a). The negative Z-score abundance and positive Z-divergence values for CSat06-26 in *Calliptamus italicus* and *Calliptamus abbreviatus* revealed the pattern of contraction due to point mutation, but it is amplified in *Calliptamus barbarus*. All the remaining satDNA families have shown the same trend of change, except CSat09-130, CSat16-225, and CSat17-223, which have gained in *Calliptamus italicus* species, representing recent amplification. Positive values of Z-abundance and Z-divergence indicate that multiple amplification events of satDNA lineages may occur, such as that satDNA from different sites is amplified (Figure 5a). The 5S-rDNA-01 and 5S-rDNA-02 divergence and abundance repeat landscape are presented in the Supplementary Materials (Figure S7).

The color-enhanced profile of the 5S-rDNA-01 revealed similar read depth coverage in the genomes of *Calliptamus italicus* and *Calliptamus barbarus*, but lower coverage for *Calliptamus abbreviatus*. The reason for high and low coverage is reflected in the variant profiles of the family. The species-specific signature of 5S-rDNA-01 is more evident in *Calliptamus abbreviatus* than in the other two genomes, where the consensus sequence from monomer position (870) onwards did not show any read coverage (Figure 5b). The color-enhanced profile of 5S-rDNA-02, as with the variant profile graphs, was identical in all three species. However, there is a 12 bp valley at the monomer site of (153–164), inferring that these base pairs were deleted as a consequence of deletion mutation. The variation in the satDNA family profiles across three species is coerced by the changes in repeat abundance and sequence divergence relative to the consensus sequence. Similar profile patterns were also observed in the CSat01-880 and CSat05-270 families. The sharp end profile of the CSat12-26 satDNA family indicates a novel spread of this family in *Calliptamus barbarus* and degenerated copy residues were also observed in *Calliptamus italicus*. All other satDNAs profile details are provided in the Supplementary Information (Figure S8).

## 4. Discussion

### 4.1. Genome Sizes and Divergence Timescale of Calliptamus Grasshoppers

Orthoptera insects have the largest genomes of all insect orders; the average grasshopper genome has been reported to be 9 Gb, with a minimum of 1.5 Gb and a maximum of 16.6 Gb. The transposable elements (TE) account for the largest proportion of the genomes [1,6]. The first two locust species documented in literature, *Locusta migratoria* and *Schistocerca gregaria*, have the largest genome assemblies, with 6.5 and 8.6 GB, respectively [46,47]. The recently published assembled genome of the morabine grasshopper is the third-largest assembled genome [48]. In light of the enormous genome size and the fact that repetitive sequences contribute to genome expansion, we conducted a comparative analysis of transposable elements in three *Calliptamus* species. The genome sizes of these species were determined using flow cytometry in another experiment. The *Calliptamus barbarus*, *Calliptamus italicus*, and *Calliptamus abbreviatus* have genome sizes of 10.37 pg, 10.1 pg, and 9.99 pg, respectively [42]. On the orthoptera phylogenetic tree, the genus *Calliptamus* diverged from *Peripolus nepalensis* approximately 17 mya; subsequent species to species divergence time is estimated to be approximately 8.5 mya [41]. The genome size differences and recent divergence chronology have been used to compare repetitive DNA sequences and anticipate the potential satellitome evolutionary effects across three genomes.

### 4.2. Repeatome Composition and Diversity among the Genomes

Due to the short time scale of divergence between these species, most of the top clusters reflect the repetitive sequences shared among all three species, with similar abundances as expected. These shared clusters include the LTR-retrotransposon lineage, LINE, Ty3-gypsy, Penelope, and other mobile elements. Similarly, despite clade-specific differences in TE content, it has been reported that the evolutionary mechanism of mammalian TE acquisition is conserved across species, possibly due to some shared characteristics [49]. A small fraction of the clusters represents the repeats that are unique to each species, especially the satellite repeats, the most dynamic part of repetitive DNA in evolution. Our results were in agreement with [47,50] that insects, especially the orthoptera genomes, were dominated by LINE and Ty3-gypsy elements. In general, the LINE and Ty3-gypsy LTR retrotransposons dominated the repeatomes of all genomes, accounting for 16–34% of the total genomes of these species, and suggesting the LTR-retrotransposon lineage proliferation and diversification among these genomes. The current research findings are consistent with previous works [6,20,48,51] that repetitive elements are responsible for genome size evolution. Likewise, most of the LINE and Ty3-gypsy clusters of these species were relatively large, which suggests the genomic amplification of these families has contributed to the larger genome size in comparison to the other closely related genus.

### 4.3. Satellitome Analysis and satDNAs Family Number Differences

It is well established that class Insecta genomes contain a wide range of satDNA families, such as 9 satDNAs families in *Tribolium castaneum* [52], with up to 16 in *Drosophila melanogaster* [53], 76 in *Pyrgomorpha conica* grasshoppers [54], 62 in the grasshopper *Locusta migratoria* [6], 45 in *Eneoptera surinamensis* [55], 29 in Ladybird Beetle (*Hippodamia variegata*) [56], 53 in *Ronderosia bergii* [50] and 4 chromosomal races of the *viatica* species (morabine grasshopper) varied from minimum 56 to maximum 92 satDNAs families [48]. Similarly, *R. brasiliensis, S. rubiginosa*, and *X. d. angulatus* have possessed the 12, 9, and 18 satDNA families, respectively [57]. Likewise, we have discovered 20 satDNAs families by using the RepeatExplorer2 tool and most of them were shared among three species of genus *Caliptamus*. A few species-specific satDNAs families of *Calliptamus barbarus* contributed to the genome size expansion, supporting previous research suggestions regarding the substantial contribution of satDNAs to the evolution of genome size, but there are also contrary findings, which indicate that satDNAs may not be a primary source [58].

### 4.4. Satellite DNA Familiies Monomer Size Variation and Double Peak Pattern

The satDNAs vary extensively in monomer size and length. In the current comparative satellitome analysis, satellite DNA families ranged in size from the smallest 26 nt CSat06-26 family to the largest-recorded satellite DNA family in the orthoptera order, CSat13-2150, with 2150 nt. Previously reported largest satDNAs in different grasshopper species were 320 bp (PcoSat25A-320) long in *Pyrgomorpha conica* [54], 784 bp (RbeSat14-784) in *Ronderosia bergii* [50], and 400 bp long (LmiSat05–400) in *Locusta migratoria* [6]. Likewise, other insect species have also been reported to have satellites with the largest repeat units, such as the ant *Monomorium subopacum* [59], which has a repeat unit of 2.5 kb, the 1169-bp PstI family in *Misolampus goudati* [60] and a 2 kb repeat unit size (HvarSat07-2000) in *H. variegate* [56]. The monomer length variation does not affect the A+T richness and copy number. There was no significant correlation observed between K2P divergence against monomer length (rs = −0.267, t = 0.270, *p =* 0.26) and A+T content (rs = 0.02, t = 0.09, *p =* 0.92) in *Calliptamus italicus* species. Unlike in *Calliptamus italicus*, the K2P divergence has shown a positive correlation with monomer length (rs = 0.477, t = 0.03, *p =* 0.03) in *Calliptamus barbarus* and in *Calliptamus abbreviatus* (rs = 0.531, t = 0.023, *p =* 0.023). The CSat01-800 family repeat showed two peaks in *Calliptamus italicus* and *Calliptamus barbarus*, one pointing to very low divergent sequences, and the other at approximately 15% divergence (Figure 4). Similarly, CSat05-270, CSat13-2150, and CSat11-220, except in *Calliptamus barbarus,* showed two types of abundant repeats in two species with different divergence, reflecting that they may have different periods or homogenization tendencies. In contrast to the other two species, CSat14-862 and CSat15-1533 have shown more than two peaks in *Calliptamus abbreviatus*, indicating the presence of more than two strongly divergent repeat units. This pattern of double peaks is not uncommon because it has been previously reported in the VspSat01-59 family in the fern *Vandenboschia speciosa*, and HvarSat01-277 in *H. variegate* both showed two types of divergent repeats on a repeat landscape [56,61].

### 4.5. Z-Score Abundance and Divergence Values and TEs Profiling

The Z-score values for abundance and divergence are significantly important to assess the overall gain and loss of each satellite repeat across the phylogenetically closely related species [62]. The Z-score abundance and divergence values revealed the amplification of CSat01-800 and CSat02-138 in only *Calliptamus abbreviatus* species, and contraction for *C. italicus* and *C. barbarus*. Similarly, CSat03-159 showed contraction in *Calliptamus abbreviatus* and *Calliptamus barbarus*, thus pointing to an ancestral contraction of this family. The CSat04-181 and CSat05-270 families have shown amplification (homogenization) only for *Calliptamus barbarus* and *Calliptamus italicus*, respectively. The negative Z-score abundance and the positive Z-divergence values of CSat06-26 in *Calliptamus italicus* and *Calliptamus abbreviatus* revealed the pattern of contraction due to point mutation. The Z-score abundance and Z-score positive values reflect that an event of amplification could occur at multiple satDNA lineages, such as satDNA from different loci being amplified. The color-enhanced profile of 5S-rDNA-02 revealed a gap of almost 12 bp in the middle of the monomer site of (153–164) in all three species, implying that these base pairs were deleted through the action of deletion mutation. Similar findings for the CharSat01-52 family with 3 bp valley at 22–24 monomer position have been reported in the genomes of *Hemiodus gracilis* and *Brycon orbignyanus* [63].

## 5. Conclusions

The comparative analysis of repetitive DNA sequences in the genus *Calliptamus* grasshoppers revealed that most transposable elements (TEs) were highly conserved across three genomes, which could be attributed to the short time of divergence (8.5 mya) on an orthoptera phylogenetic tree. The evolutionary changes of satDNAs across three genomes, on the other hand, reaffirmed the dynamic nature of satellite DNA, which is the main cause of genome size variation. For some satellite DNA families, the Z-score abundance and divergence values reflect the amplification and contraction processes. The color-enhanced profiles of satDNAs and rDNAs demonstrate the action of deletion mutation in some repeat families. Understanding the structure and composition of genomes is now critical not only for learning about their past evolution but also for anticipating their future evolution.

## Figures and Tables

**Table 1:** The table contains the divergence%, abundance%, copy number for *Calliptamus italicus, Calliptamus barbarus,* and *Calliptamus abbreviatus* estimated by using Repeatmasker, as well as A+T percentage of each satDNA families.

			*Calliptamus italicus*				*Calliptamus barbarus*				*Calliptamus abbreviatus*			
Repeat name	Length (bp)	A+T%	R.Length (bp)	K2P Divergence	% Abundance	Copy Number	R.Length (bp)	K2P Divergence	% Abundance	Copy Number	R.Length (bp)	K2P Divergence	% Abundance	Copy Number
CSat01	880	51	1,868,736	8.09	0.2377	2679288	2,524,598	12.26	0.3211	3566082	2,914,794	3.21	0.4716	5005862
CSat02	138	52	2,047,691	9.55	0.2605	18721455	1,923,378	4.11	0.2447	17324765	2,856,892	7.84	0.4622	31287328
CSat03	159	52	2,086,105	3.1	0.2654	16553632	689	7.8	0.000087	5386	216,925	9.46	0.0351	2061893
CSat04	181	59	589,404	4.79	0.0749	4108551	1,542,589	5.83	0.1962	10593841	37,444	15.15	0.0060	312649
CSat05	270	58	841,952	10.69	0.1071	3934393	623,587	11.31	0.0793	2870880	504,540	11.81	0.0816	2824135
CSat06	26	54	8891	18.61	0.0011	431451	917,670	4.44	0.1167	43872762	55,901	20.11	0.0090	3249376
CSat07	1544	51	126,042	6.2	0.0160	102996	38,987	7.91	0.0049	31387	207,484	3.69	0.0335	203091
CSat08	294	53	65,871	6.15	0.0083	282683	26,312	19.21	0.0033	111247	95,366	5.64	0.0154	490230
CSat09	130	61	2,457,238	11.92	0.3126	23848338	1,984,831	12.19	0.2525	18978504	1,865,417	12.15	0.3018	21686343
CSat10	1246	55	1,147,659	6.55	0.1460	1162114	1,280,293	6.43	0.1628	1277241	2,613,887	4.83	0.4229	3170461
CSat11	220	59	439,785	8.85	0.0559	2522156	513,195	7.75	0.0652	2899622	586,905	11.32	0.0949	4031799
CSat12	42	39	681	10.09	0.000086	20457	598,775	7.43	0.0761	17721324	NA	NA	NA	NA
CSat13	2150	49	261,807	15.46	0.0333	153637	279,754	15.42	0.0355	161740	271,878	13.69	0.0439	191112
CSat14	862	52	61,717	8.68	0.0078	90334	60,903	10.53	0.0077	87824	91,750	9.6	0.0148	160861
CSat15	1533	53	61,281	8.14	0.0077	50435	93,124	9.26	0.0118	75509	150,566	7.78	0.0243	148435
CSat16	225	58	156,325	5.51	0.0198	876597	11,758	6.42	0.0014	64958	90,354	9.05	0.0146	606902
CSat17	223	59	206,411	7.01	0.0262	1167837	5982	7.19	0.0007	33344	64,944	7.84	0.0105	440137
CSat18	185	60	70,952	9.64	0.0090	483890	81,286	9.32	0.0103	546167	78,435	9.49	0.0126	640754
CSat19	897	53	41,687	3.73	0.0053	58635	34,831	11.18	0.0044	48267	128,261	2.5	0.0207	216100
CSat20	245	60	NA	NA	NA	NA	103,174	13.21	0.0131	523462	NA	NA	NA	NA
Total					1.5954				1.5957				2.0763	
Mean				8.566315789				9.262631579				9.175555556		
SD				3.828965933				3.767555709				4.495353447		

## Data Availability

The data underlying this article are accessible at Sequence Read Archive (SRA) of the National Center for Biotechnology Information under BioProject PRJNA638780 https://www.ncbi.nlm.nih.gov/sra?linkname=bioproject_sra_all&from_uid=638780 (see Supplementary Table S1).

## Supplementary Materials

The following are available online at https://www.mdpi.com/article/10.3390/insects12090837/s1, Table S1: Genome data information used for TEs analysis, Figure S1: The graphical summary of individual clustering analysis of the genus *Calliptamus* species. The heights and widths of the bars denote superclusters, corresponding to the number of reads in the superclusters on *y*-axis and their proportions in all examined reads on *x*-axis. Individual clusters are represented by rectangles inside the supercluster bars. The proportions of clustered and single reads are shown in the blue and pink background panels, respectively. On the left of the dotted line are the top clusters. (**a**) *C. abbreviates*, (**b**) *C. italicus*, (**c**) *C. barbarus*, Figure S2: The proportion of annotation of classified and unclassified clusters by RepeatExplorer2. Clusters are arranged in ascending order, Figure S3: The classification of *C. barbarus* species-specific unclassified cluster CL-222 and CL-170 using the YASS tool. The CL-222 graph depicts a typical layout of tandem repeats, and the contigs in this cluster were compared to one another (Self-comparison). The diagonal lines (green lines) in the graph represent the tandem repeat, which allows us to characterize it as satellite DNA repeat CSat20-245. The other cluster CL-170 has not shown any evidence of tandem repeat and left it as unclassified, Figure S4: The Spearsman rank-order correlation test. There was no significant correlation observed between K2P divergence against monomer length (rs = −0.267, t = 0.270, *p* = 0.26) and A+T content (rs = 0.02, t = 0.09, *p* = 0.92) in Calliptamus italicus species. the K2P divergence has shown a positive correlation with monomer length (rs = 0.477, t = 0.03, *p* = 0.03) in *Calliptamus barbarus* and in *Calliptamus abbreviatus* (rs = 0.531, t = 0.023, *p* = 0.023), Figure S5: Satellitome comparative line graphs with single-peak and flattened distribution of abundance against the divergence, Figure S6: Satellitome graphical-structures for each family of satDNAs. The comparative graphical structures of satellite DNAs and rDNA-repeats reported in the Repeatexplorer2 output. Colors in the graph represent the species-specific reads (green-for-italicus, blue-for-abbreviatus, and red-for-barbarus) where the node denotes a specific read and the edges as a bridge (connecting lines) between the similar reads, Figure S7: Interspresed repeat landscapes and line graphs of rDNAs and CSat20-45 family. The interspersed repeat landscape of rDNA-repeat, CSat20, and unclassified cluster has been shown here (**a**). In *C. barbarus*, a distinct individual repeat landscape demonstrates the existence of a single abundant peak of CSat20-245 satellite DNA. Similarly, the recent abundant copy of 5S-rDNA-01 in *C. italicus* and *C. barbarus* revealed one peak and multi-variant flat distribution in *C. abbreviatus*. There are two peaks of 5S-rDNA-02, the recent one and another ancient/degenerated highly divergent copy (**b**), Figure S8: The individual satellite DNAs repeat and variant profiles demonstrate the strong species-specific signatures. Species profiles arranged in a sequence of *C. abbreviatus* => *C. barbarus* => *C. italicus*. Most of the satDNAs profiles have shown reasonable read depth coverage with the decrease of variation in the variant profiles, which are represented in dark red color. The CSat12 profile uneven coverage of repeats with sharp boundaries shows the differential amplification of fragmented copies of this repeat, implying the novel spread of satellite DNA sequence. Additionally, some profiles suggest the residual existence of some of the satDNAs degenerated copies such as CSat12 profile in *C. italicus*.
