# Supplementary material for: Comparative Analysis of Transposable Elements in Genus Calliptamus Grasshoppers Revealed That Satellite DNA Contributes to Genome Size Variation
Source: Insects. 2021 Sep 17;12(9):837. doi: 10.3390/insects12090837 (PMC8466570; doi:10.3390/insects12090837)
Supplement: Supplementary file 1 [file insects-12-00837-s001.zip › suppl.pdf]

Table S1. Genome data information used for TEs analysis.

Species Name	Collection site	BioProject Number	SRR Number	Genome Sizes (pg)	NGS Data used in Repeatexplorer2 (reads)	Individual Clustering (coverage)	Comparative Clustering (coverage)	No. of clusters*	No. of Super Clusters*
C. abbreviatus	Changchun, Jilin, China	PRJNA638780	SRR14826315	9.99	16,283,700	0.5x	0.03x	345	292
C.barbarus	Alxa Zuoqi, Alxa League, Inner Mongolia, China	PRJNA638780	SRR14826314	10.37	16,903,100	0.5x	0.03x	355	320
C. italicus	Zhangye City, Gansu Province, China	PRJNA638780	SRR14826313	10.1	16,463,000	0.5x	0.03x	479	453

***Clusters and Super Cluster above the threshold of 0.01%.**

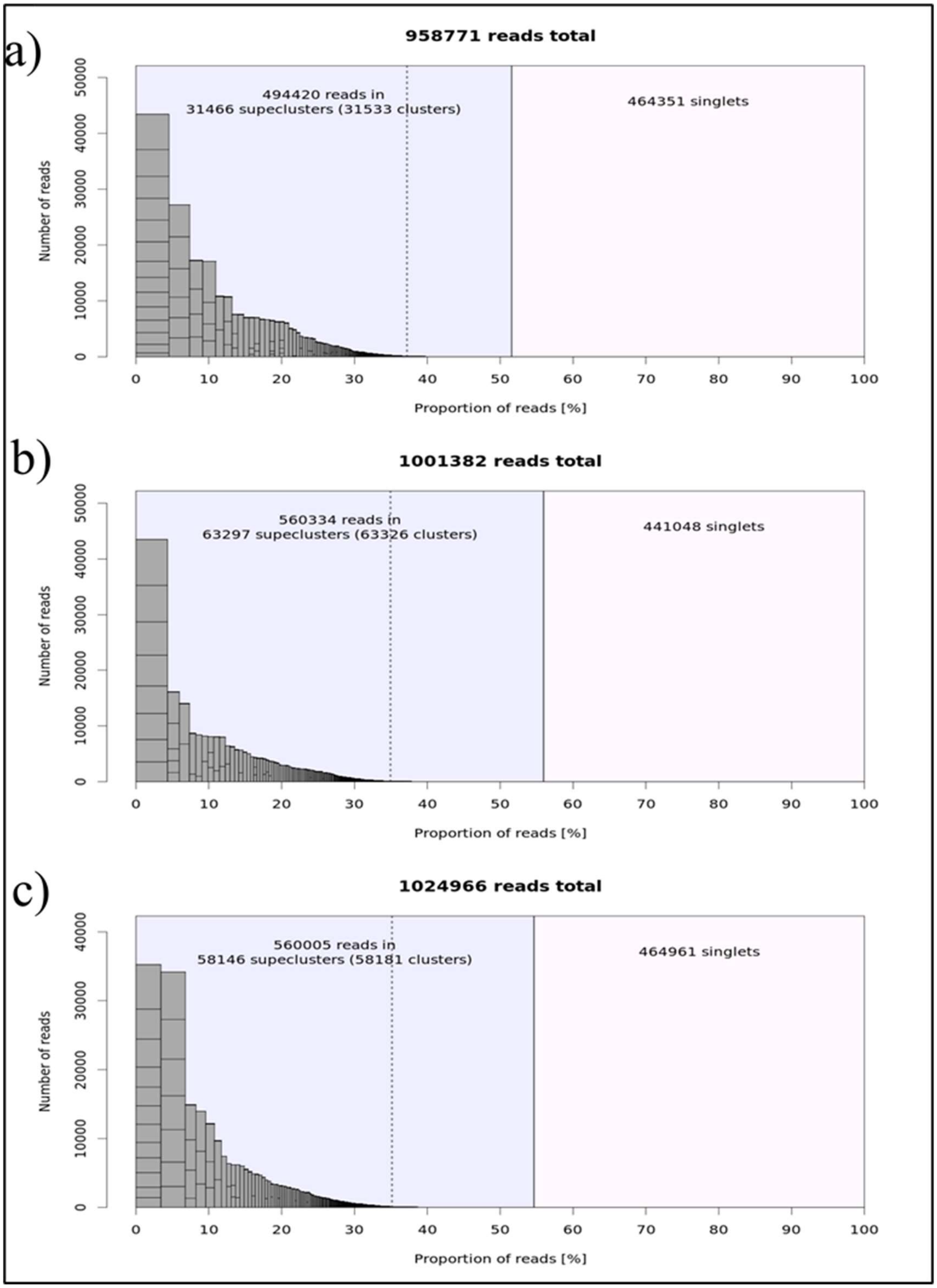

Figure S1. The graphical summary of individual clustering analysis of the genus *Calliptamus* species. The heights and widths of the bars denote superclusters, corresponding to the number of reads in the superclusters on y-axis and their proportions in all examined reads on x-axis. Individual clusters are represented by rectangles inside the supercluster bars. The proportions of clustered and single reads are shown in the blue and pink background panels, respectively. On the left of the dotted line are the top clusters. **a)** *C. abbreviates,* **b)** *C. italicus*, **c)** *C. barbarus.*

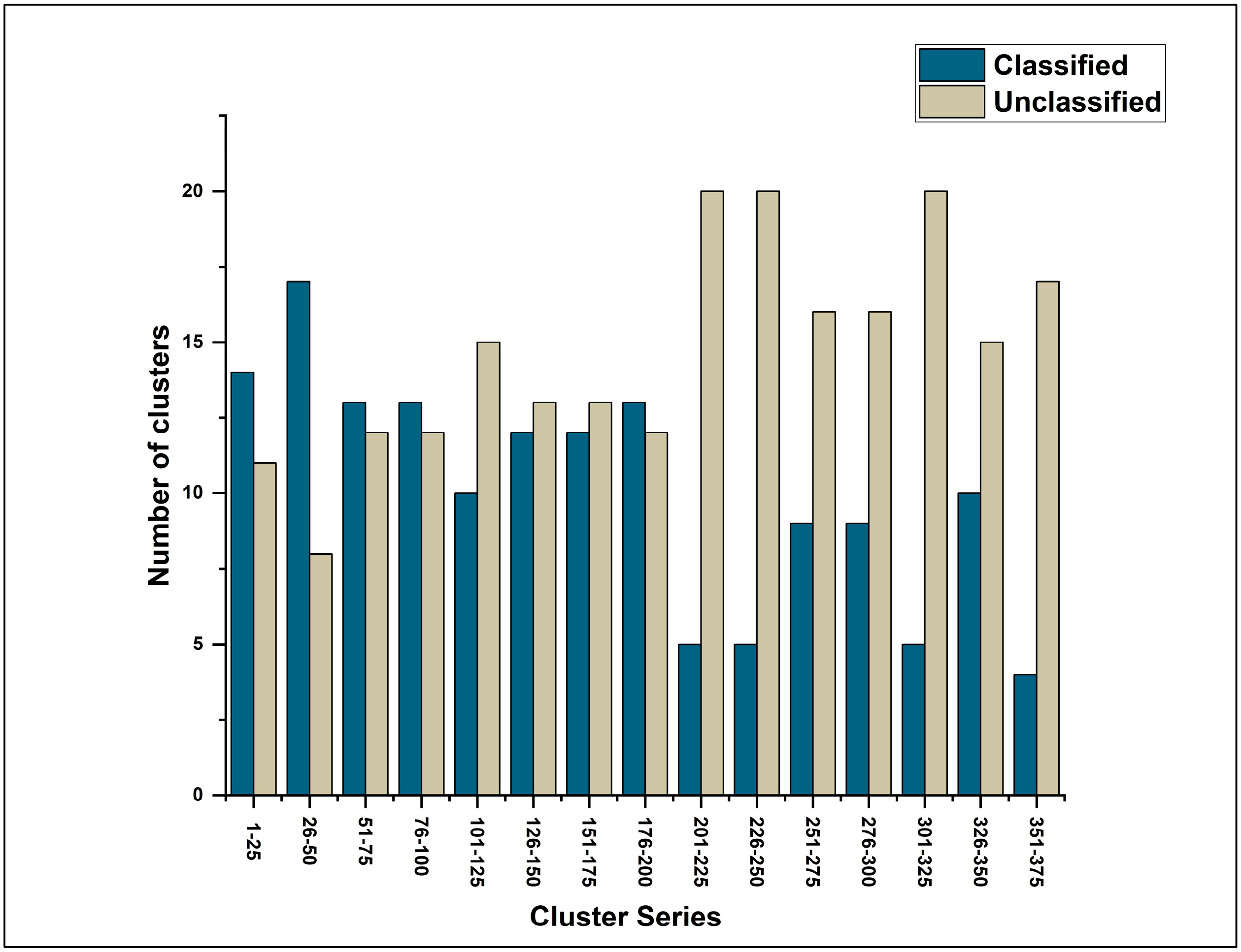

Figure S2. The proportion of annotation of classified and unclassified clusters by RepeatExplorer2. Clusters are arranged in ascending order.

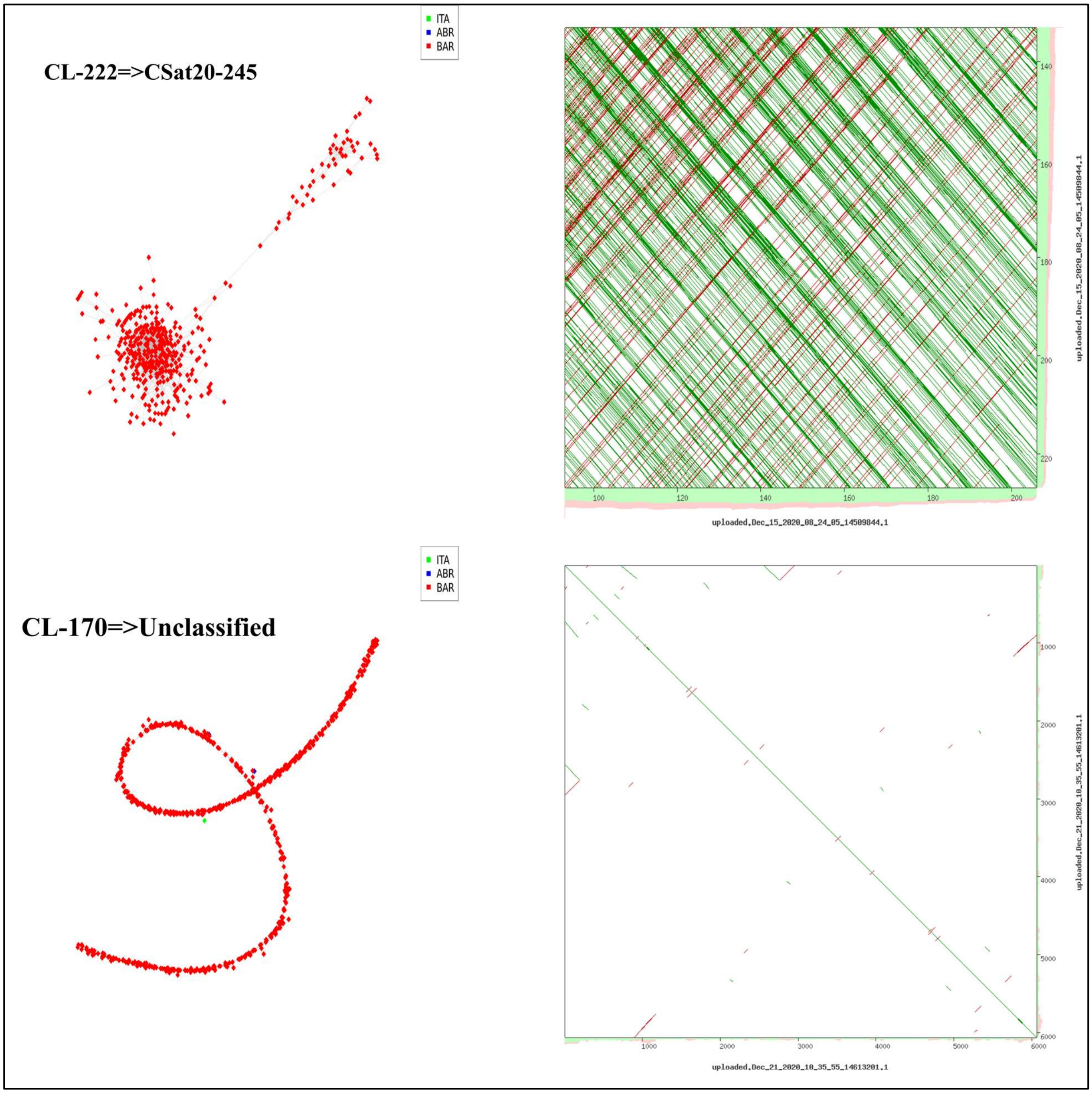

Figure S3. The classification of *C. barbarus* species-specific unclassified cluster CL-222 and CL-170 using the YASS tool. The CL-222 graph depicts a typical layout of tandem repeats, and the contigs in this cluster were compared to one another (Self-comparison). The diagonal lines (green lines) in the graph represent the tandem repeat, which allows us to characterize it as satellite DNA repeat CSat20-245. The other cluster CL-170 has not shown any evidence of tandem repeat and left it as unclassified.

C. italicus

Correlations

			Length	Abundance	K2P
Spearman's rho	Length	Correlation Coefficient	1.000	-.100	-.267
		Sig. (2-tailed)	.	.684	.270
		N	19	19	19
	Abundance	Correlation Coefficient	-.100	1.000	-.067
		Sig. (2-tailed)	.684	.	.786
		N	19	19	19
	K2P	Correlation Coefficient	-.267	-.067	1.000
		Sig. (2-tailed)	.270	.786	.
		N	19	19	19

Length vs abundance (rs=-0.1 , t=0.684, p= 0.68) , Length Vs K2P (rs=-0.267 , t=0.270, p= 0.26)

K2P vs abundance (rs=-0.067, t=0.786, p= 0.78), Length vs A+T (rs=-0.322 , t=1.40, p= 0.17)

C. barbarus

Correlations

			Length	Abundance	K2P
Spearman's rho	Length	Correlation Coefficient	1.000	-.233	.477*
		Sig. (2-tailed)	.	.336	.039
		N	19	19	19
	Abundance	Correlation Coefficient	-.233	1.000	-.086
		Sig. (2-tailed)	.336	.	.726
		N	19	19	19
	K2P	Correlation Coefficient	.477*	-.086	1.000
		Sig. (2-tailed)	.039	.726	.
		N	19	19	19

*. Correlation is significant at the 0.05 level (2-tailed).

Length vs abundance (rs=-0.233, t=0.336, p= .31), Length Vs K2P (rs=0.477, t=0.03, p= 0.03)
K2P vs abundance (rs=-0.086, t=0.726, p= 0.70), Length vs A+T (rs=-0.322, t=1.40, p= 0.17)

C. abbreviatus

Correlations

			Length	Abundance	K2P
Spearman's rho	Length	Correlation Coefficient	1.000	.311	-.531*
		Sig. (2-tailed)	.	.196	.023
		N	19	19	18
	Abundance	Correlation Coefficient	.311	1.000	-.294
		Sig. (2-tailed)	.196	.	.236
		N	19	19	18
	K2P	Correlation Coefficient	-.531*	-.294	1.000
		Sig. (2-tailed)	.023	.236	.
		N	18	18	18

*. Correlation is significant at the 0.05 level (2-tailed).

Length vs abundance (rs=-0.311, t=0.196, p= .44), Length Vs K2P (rs=0.531, t=0.023, p= 0.023)
K2P vs abundance (rs=-0.294, t=0.236, p= 0.23), Length vs A+T (rs=-0.322, t=1.40, p= 0.17)

Figure S4. The Spearsman rank-order correlation test. There was no significant correlation observed between K2P divergence against monomer length (rs = −0.267, t = 0.270, p = 0.26) and A+T content (rs = 0.02, t = 0.09, p = 0.92) in Calliptamus italicus species. the K2P divergence has shown a positive correlation with monomer length (rs = 0.477, t = 0.03, p = 0.03) in Calliptamus barbarus and in Calliptamus abbreviatus (rs = 0.531, t = 0.023, p = 0.023).

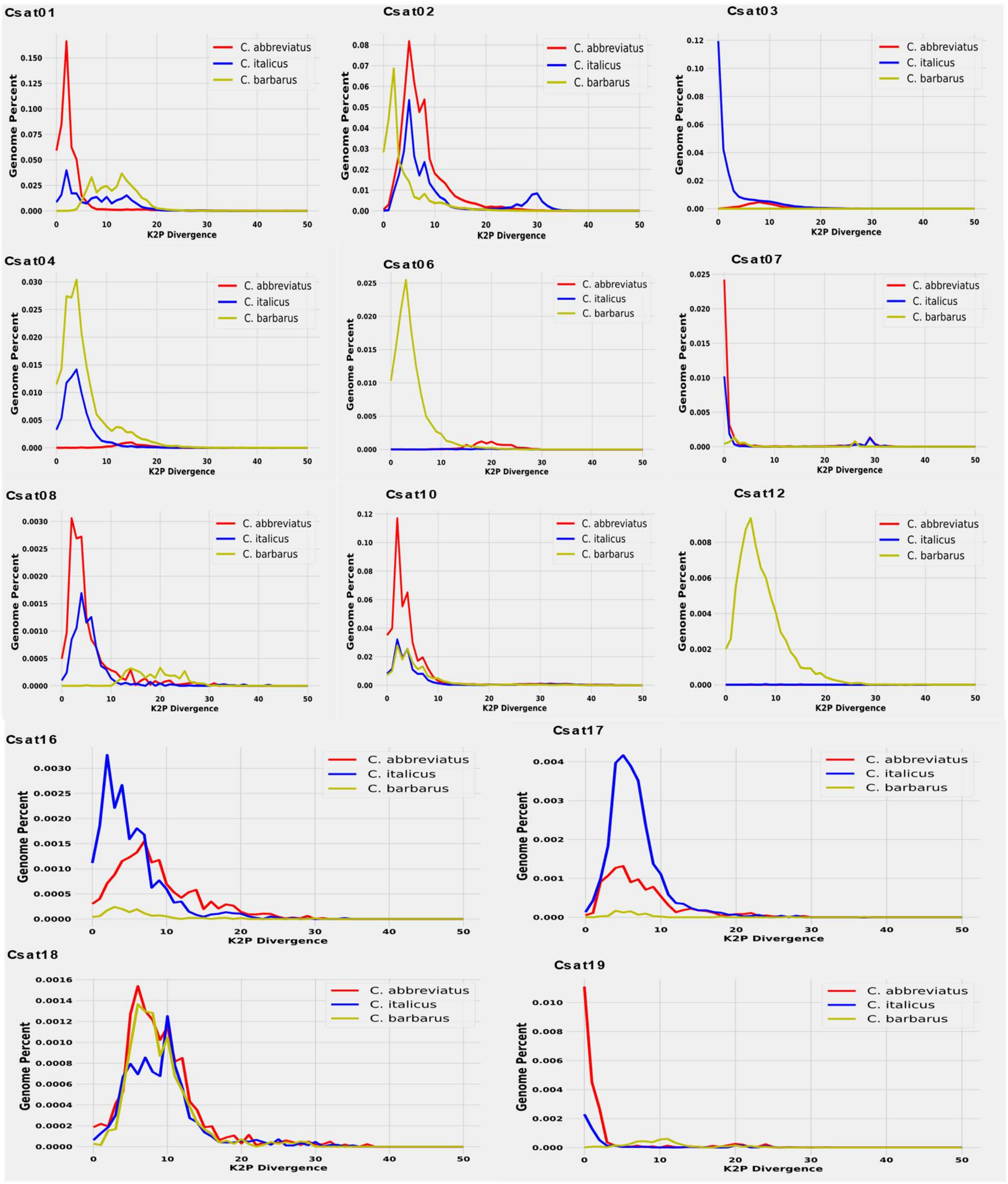

Figure S5. Satellitome comparative line graphs with single-peak and flattened distribution of abundance against the divergence.

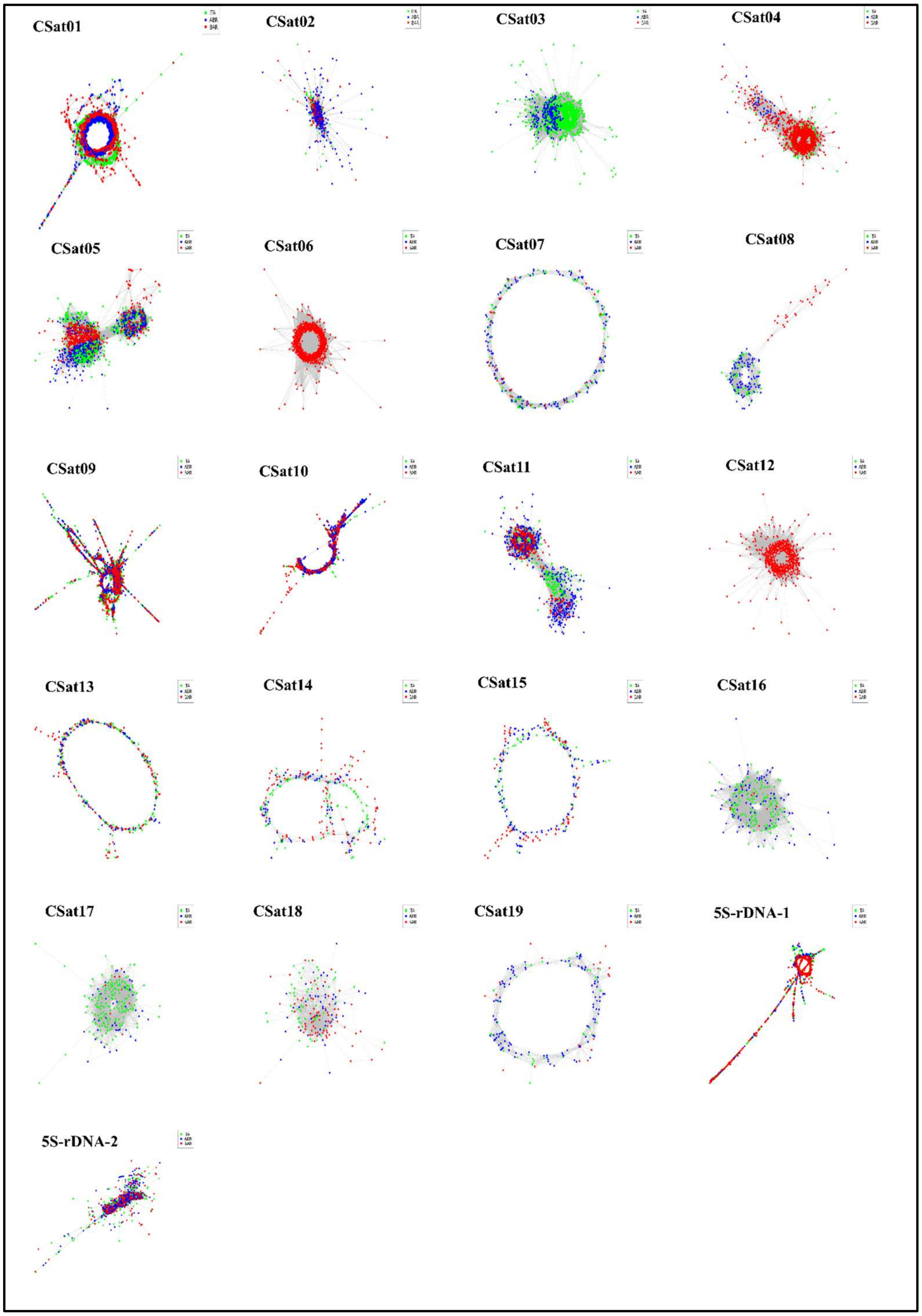

Figure S6. Satellitome graphical-structures for each family of satDNAs. The comparative graphical structures of satellite DNAs and rDNA-repeats reported in the Repeatexplorer2 output. Colors in the graph represent the species-specific reads (green-for-italicus, blue-for-abbreviatus, and red-for-barbarus) where the node denotes a specific read and the edges as a bridge (connecting lines) between the similar reads.

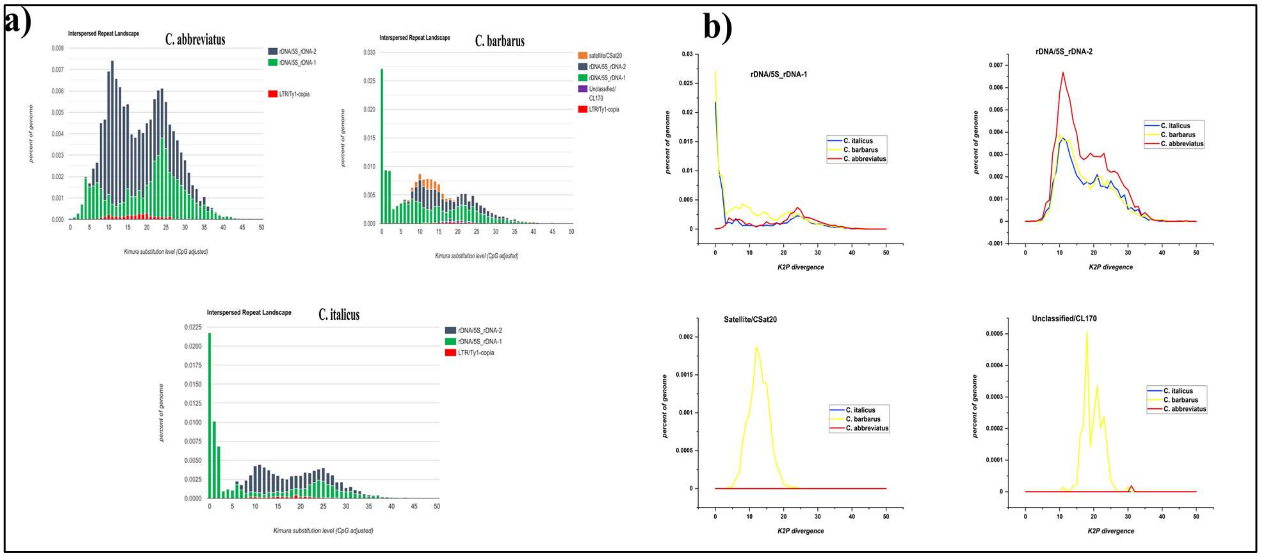

Figure S7. Interspresed repeat landscapes and line graphs of rDNAs and CSat20-45 family. The interspersed repeat landscape of rDNA-repeat, CSat20, and unclassified cluster has been shown here **(a)**. In *C. barbarus*, a distinct individual repeat landscape demonstrates the existence of a single abundant peak of CSat20-245 satellite DNA. Similarly, the recent abundant copy of 5S-rDNA-01 in *C. italicus* and *C. barbarus* revealed one peak and multi-variant flat distribution in *C. abbreviatus*. There are two peaks of 5S-rDNA-02, the recent one and another ancient/degenerated highly divergent copy **(b)**.

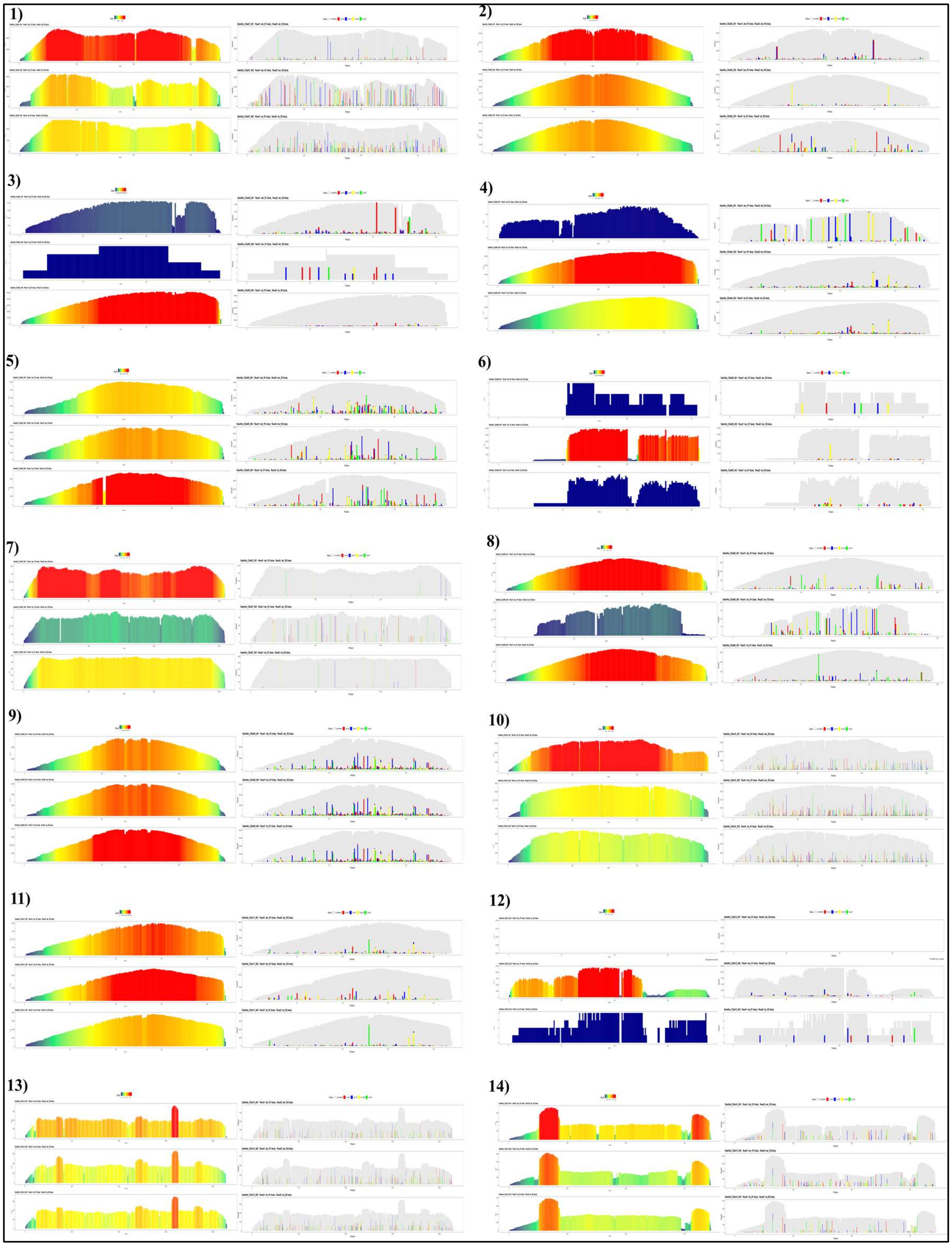

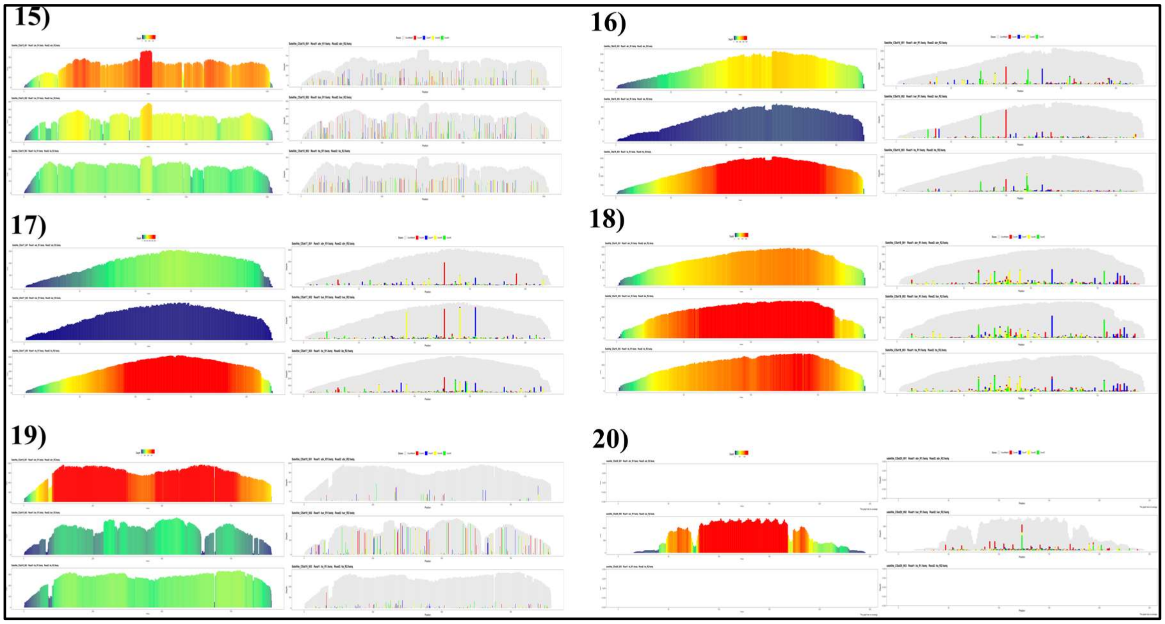

Figure S8. The individual satellite DNAs repeat and variant profiles demonstrate the strong species-specific signatures. Species profiles arranged in a sequence of *C. abbreviatus* => *C. barbarus* => *C. italicus.* Most of the satDNAs profiles have shown reasonable read depth coverage with the decrease of variation in the variant profiles, which are represented in dark red color. The CSat12 profile uneven coverage of repeats with sharp boundaries shows the differential amplification of fragmented copies of this repeat, implying the novel spread of satellite DNA sequence. Additionally, some profiles suggest the residual existence of some of the satDNAs degenerated copies such as CSat12 profile in *C. italicus.*

The detail of consensus sequences of the satDNAs and 5S-rDNAs identified in the current experiment.

>CSat01#Satellite/CSat01

gtaataggcagccactaagcagagtgtcgcagtggaagcttactgggaccataactcacaggtccgtggatcgaaaccaagcctataagt
atcgtgcgcaatccagttgtgccactggggctacagcacagccttcccaaacagccatattcacattaaatccgtcgtgtagcgaccagaaat
acgtcatctatcgtcatttacggctgtactgagacttcggcaactgcctacgaatgattcacactactctactttggtaacaacgattacttgcaa
atggacagcttttagtcacggaatacgtgcagaaaaccgcgcgccttgtcgtttgctaactgacacacagcgaaacaaggtaggtggcgtg
ttctaagttctgaaggagcacagcgaatgcgcatctccagcagaggcgctttccttgggtgctgccagtagggcgcagaaggccagagg
gaagatgctatcgttgccaatggacaaaaggaaaacaaaaagcagaaaaaaatccttccttgtgtagcaacacttttctgcgagactgagg
agaaagcagtggccctgtgacgcaacggataacgcctctgaccacgggcagaagattccaggttcaaatcctggcagggtcgtaattttgt
taattggtggagcgttgctaggcaacggacctggccatcttactgctcttattcccacggtatttcgcagttgcaagccttgctgatcacgata
aataccgcctcccgcaagcgtcagcgtagcgtcagtcgagcaaagtcgagccaagtcaagctgagagctgtaggaaatgctgcatctttaa
atttagatatataaaacatcgcagggaagacttctcatgagttccgtgtcagag

>CSat02#Satellite/CSat02

gaatatagtataaacagcattggcgattcagtgaaatttccgaactttcgtcgtgtctaaacgacttgcgaagcgtttggcgctcatggcgca
cctcagcagccactgggcagtgctgggacaaccaaagaatcgatttagtataaacagcattggcgattcagtgaaatttccgaactttcgtc
gtgtctaaacgacttgcgaagcgtttggcgctcatggcgcacctcagcagccactgggcagtgctgggacaaccaaagaatcgattgaata

>CSat03#Satellite/CSat03

gttagttgatttcaatcgagatgcgaagagcaggattatgatgggaagcgttttgcgggcatggggcaactcagtaggcccgggatagtg
tggggacaaccgtagaatcgatcgaatgcaggtaacaggagcactggcgatggtctgaaatttctaaat

>CSat04#Satellite/CSat04

gcttcctaagggaaacgtccttctgtgtgtgctgactggtctcgtgaacaatttgcacatattgtcgttgaatacagtgctaaattaatgtaaag
ccactgagctacaccgggattttcatacatatccagacctgtatcgacaaaatcaactgtttcggagagtctcgtaatttgcgttc

>CSat05#Satellite/CSat05

cctctccaaaagtatttcatcgatacaaaaagtaggtgtttcatatcagcatccatccagtagcgacagtgtgacgtcttgcagagttaatttcc
gtttcgtattagttttacaagcaatcgacgactgagaaataacattgtaagctttgtgtaacttctgtccaaaatctattttgtgttgtttaacgcct
atggcattaaacataggttgaaaattccccttaaggggaaatggggcctcctccccttacagcccgcccttt

>CSat06#Satellite/CSat06

gtcgaggatttggctaccttaagagacttaagagagtcgaggatttggctacgatttggctaccttaagagagtcgatgatttggctacctta
agagagtcgagcgaggatttggctaccttaagagagtggatttggctaccttaagagagtcga

>CSat07#Satellite/CSat07

actctgtcacacaccacgaaagtttttcataacgcaggcgttgactacaacagcttcccttccacagacgacttatccgactttgcacgacgct
cacgcagcgctcacgctagcgacatcctctcttaaaacttcacgtcgagcctaaaaatatgagcacgtatctcctaggacacaggccaccctc

ctaccgttggtgcacgatgtgtggagtcagcgcagggccgcaggctttcactgttcacgcctacaaaacctctgctcgcttgtaaagagctg
aaaaaaatcgcgagactcccatgtcgctacacgatttcattggcacgacatctgccgtcaggtgtctcaaccatcaacatcagcagctcccga
atggacgtgaaaaaaagaaagctgtactggagatttcattgcttcggcacaaggcagttcttttcgacgcagcttgcagtatcttatggaagt
gcttgtactgctcctaaagcaaattttggggaacaatggagggtcgttgctgcacataataatgcatttactcggagttcagatccattgcagc
cgctaagccttcgcaactgcctttctggtcaccagcaccgtctccactggaccttattgacagctcattgttctacatgctcgccagtccatgtgt
tttaaaaacgttggagacgccgggtatcgatcccggtacctctcgcatgctaagcgagcgctctaccatctgagctacgtccccacactgcg
atgtgtagcctcggaaggtcaattcagtgacaaagatacaactactgccttcattccgcagacagccactaatctcaacaagcctcgtgaag
acggcgacaagtgttgactgcactttagacccagtcccagatggacgtagtcagagtagcactcactatgtcgaaaatgattctaaggccgt
tcgctgaataaagctacaaccttgcctgctcgatgttaagctgttacacctagagacgtttcaccttgcaggcacgtcgagtgctgaaaatcct
ttcatacacagctcacatcacctcgcctcagtgtccatcctgtgtgattctgacgctgctttggacatttcgcgtgattacgagaaggtcgacaa
cttacgcttaccaccaagggagtacaaaatttggaggcgccggggcttgaacccgggacctttcacatgcaaagcgaacgctctaccagct
gagctacgcccccacgtgctagtaaacgctctggtgtaccctcctctgttcttctactctgcactaacagacgttcacacagacagtatgtctcg
ttgctcacataaaataagtacaacttttgccagctgttacgtcttacagtgaaggtacttggcatatcgctcctttctcgttccctgtcgacaatca
cacagcaagaaagtaaacacaagccaatctcaagtcggtcgtgcacctgactgtgcatatcaactagctgcctcacaggtttctactgcgtac
cacgccggtcttggaactacatatgggagagcttcctccattttcacg

>CSat08#Satellite/CSat08

tatacagctcacgtggtgtagtgcgtgcgttccgccacccatttacctgaaatgcaagctccagacatgacctggcgtaagaaggaacgtgc
tttatttgagcaggcaatacgcttccaccattcgacttaaactggtcccctcccagtcataactgatgaactcgtgtaatcaatgtgggcacaca
tattggtcatctaatgcagagctgtgttttccaatgtgtgtttttcatggcgggaaccaatacgctgcctgtcttcaaatctaccacattctgccac
ctacccattc

>CSat09#Satellite/CSat09

tacggatttcgacagaatccacagttttattccgatcttgatgaaattttgcacacttgaccttcaaaacaagaggaaggtcactgtctacataa
aatttcgtatggtgcatggcggagggtactttacttacggacttcgacagaatccacagttttattccgatcttgatgaaattttgcacacttga
ccttcaaaacaagaggaaggtcactgtctacataaaatttcgtatggtgcatggcggagggtactttact

>CSat10#Satellite/CSat10

taaccgtttaaatagtatttaccctgttcatttgaactcttggaataacattgtattagatcgccacacctaattcagggatatacacgtctcacag
tattggagccaacattttgagtgaaatgctaaaatatgtgtcagattctgtttctgcgttctcgttgtataatgctaggactggcagggaagta
agagcctccttaagtactaggaaagatctttcttgcgcctcattccagtaaaacttggcatctccctgcagaagttcttgcaatggacgtgcctt
gttacagaaatcttttatgaagcgtcggtagtacgagcacatcccgaggaagcttctcacatcacgaatgtgtcggctagttggcaattctgt
gactgcccttattttctctgtatcaggacggactccatctccgttaactaggtgccccaggatttttatctcttgggcgacaaataggcgcttcct
cggattcagacggagacatgcagtttgaacacacttcaacacagctgacaggcgactcagatgttcttcaaatgttttcgagaaaacgacaa
atcatctagatagcaaagacacgtcgtccacttaaggtgtcgaagcaagtagtccatcatgcgtttgaatgtggccggagcattacatagtc
caaatagcattactttgaattcatagaggccgtcaggtgtaataaaagccgtcttttccctgtcagcttcgtcaacctcgatttgccaataacct
gtttgcatgtccatggttgagaaatacttagcaccattcaagcagtctaaggtgtcgtcaatacgcggcagtgggtagacgtctttctttgtga
ctttgttcagccgccgataatcgacacagaaacgccacgtgccatatttcttctttacgaggaccactggagaagaccaaggactctctgag
ggttgaataatgtcgtcgtgcagcatcttctccacttcttcccggtcattcgtcttccagctggtgacatcctatacgaatgctagctaattgtag
gatgatccccagtgttgatacggtcctttaccgtgggctgttttgcatggctcttctcctgctgggctttgaatgcacccgaaaattggcgtagt

atagctaacacctgctggcgttgttccttggtcaggctgggccctattggaatttccacagttgtcccctccagcgtgttgtctgtattaacaac
agagcatgggtcttcgtgaa

>CSat11#Satellite/CSat11

tattttgattgcagcagccgctcccccagcgaagaggacgccagtggactacgatcaagacacccccacaccaaaggcttctttgagatgtg
ggcaagggaagaaattttccttcattttggtaagtatttttttgacgtttctaagtgtatataattcactcagtgcgtacactaggagatatctaca
aaaaatggaagcttaattaacattttcatgcc

>CSat12#Satellite/CSat12

ggggcggttgcaactgcttctggggcggttgcaactgcttctcaactgcttctggggcggttggcttctggggcggttgcaactgcttctgg
ggcggttgcaacttgcttctggggcggttgcaacggttgcaactgcttctggggcttctggggcggttgcaactgtttatggggcggttgca
actgc

>CSat13#Satellite/CSat13

cttggacgctgttgagtggacaggtgaaacgcactgccacctcgtttgcctctgagtacgttaaggcgtgctgtgcagccactgtgaaatga
aggagtgtgtcgtgtgcctgtgtcgtcacctcccctcgtgtctctcagtcgattttgatgcttgtcccttgatataggccgaactgagagcgtca
gctctcgcgtcagctctcgcaaagtcgagacaagtcgagacacatatacacactacgggccgcactgcaatgagttgtcgggggaaaaac
gttaattttaagggcgcgtcgcagacgtgttcatacgaaaaaatgaaggcatgttgcggtggccgggaatcgaacccggatcaactgcttg
gaaggcaactatgctgaccattacaccaccaccgcacggcgcggaagagagttacgccgaggcgtatcggcttcccgcctgccagcagc
ggccgaagttgatcgtagctgtaggcgcattacgatgtaggtgagcacatccacacacattcggacaacgcctttacgcggattttgcgtctt
ttcacgacagacgccaccgacgccgactcagaggatcgcgatgtagcgtcattgtaacgagtacactgcaggactctgtttcaatagcgct
gttttcgggaggataagacagcgctaggcggcagtcccactttcactgcaaacgactgctgaggaagcaccaggaagctcatctccagcc
gagcatcggtggttcagtggtagaatgctcgcctgccacgcgggcggcccgggttcgattcccggccgatgcatcattttatagtttctgca
acaattcggccgcatgcttacttatttgagctgcacgtgcttcaattatgtccaccgggactcgctgccagaaaaaccaaacatgctgcacgc
acgagacgttgctggacgctggcgccgaaatgtcagcagactatggtcggtctggcgtcacgagttgcagtgtccgcttggtgcctggctg
atgtggcgtaacggacaagcggagctgccttcggtaggaaaaatcgcagctccattttaacctgctgagcagacaatgtgcatgtagcagc
tgaagacaccattaaggaagcaaaactgccctaatttagagtacttaccaaatttctagaagcgctgcaagccaggtgcctcggtagcgca
gtaggcagcgcgtaagtctcataatcttaaggtcgtgagttcgatcctcacccggggcatttaatttagtctcgatcagcgccgaattcacgg
cgttgctgttgctaggaaacgatcttatttgttgtttgtaacccacgagttttgcagcaccaaaacgagaaaattattttgcctccagctgttact
accttgcttcatctcctccgctaggaatacacgatgccaaaagcgatgggtctttttctgtgttcttcgacatttagtatacgtaaaggttctcac
aaaccgagaggcgcgccgacgccagactgcagattggacacgaaatacgcatggagtgcgtctaaacgagacgttggaagtgcgtctcc
ggtgtggtctagtggctaggatacctggctttcacccaggaggcccgggttcgattcccggtaccggaacagaatttttcccgcacacaact
cgtagagttttagcccttcgaaataccgtttctggtcctcctcgtaggattagtatgaggacctgaataacaggagaagagcgctgctatgcc
attggacagcaacatgcaacgtgcttcttctgtatcgcagcaagggtctgtttcacgaggcccgttagctcagttggttagagcgtcgtgcta
ataacgcgaaggtcgtgggttcgatcccccacgggccacgtcgatttttctattgaaaaaccaacgccgatttcgacaggcaaattggtg
gccaaatgtcattacttttgtgtggaactgagacaggatccgatcccgatttgtcgagacacgcttcagccttcatccttcaacggccatatcac

tttgaataccctgtttctcgtccgatctccgctattagcaactttgggccttgtcattatttgggtgtgcgacagccggggaacacaaagtgctg
tcgactgcattaacttatttgcttcat

>CSat14#Satellite/CSat14

ctccttcgtagcagctagtgttgtagcgatgaaagcacttcggaaaataacgcaggtacatcgcttaatgtcaacatattgctctaaactgca
gcaaatgcatacgtctaaagactgttcgtcagcaagcagtcgtggccgagtggttaaggcgtctgactagaaatcagattccctctgggag
cgtaggttcgagtcctaccggctgcgttcgattttgaataaaaacaagcagatattttcgcagaaatttgaaagatgccgataacaggtgga
agcaggccactgatgcagttaccaacaagacgcggatttctgtttaggaatgtttatttagttcacgacggccgctccttctgtacagccatcg
gttcacctcgcaccaagcgtacgacagcgccattggactaggacggcagagcgccagcactgagccacgaggccccagtcgtttcgtcga
gccgtctcggtgtttgtaaacacgcaaggtgctgcatgtactagaaagtgtagtgggcagcgatcactcattactgttcttaaatttgcgttgc
attctttcgattacgatttccgttatcaggcagcgtttccttgtcttatagatctcgcagctacttggaagtgggtccatcatttaagatcccgcaa
actagcgtcagcgtggcgtcagtgggaagtctgtgaagtcgccagtggagccagaagccagtaattgcaatactacgcattcacacacac
atgtgagttggcatgcactccacaatcgccgtgtgtgagaccggatagctcagtcggtagagcattaggcttttaacctaagggtccagggt
tcaagtccctgtccgggcgaagattttaa

>CSat15#Satellite/CSat15

gaactgtgatgtagcaaaattatcggcacagcgccgttcgcgagagttttggctgacacctccacgtctaagacaggatatgactgctacac
tgtaagtatgtttaaaatcgagtaaacgtgtttgttaatggcaccactacttccgtaggtctgcattcagtttgctcacctctcagagaccttttgc
aatgaaacaaaatcacttgcagaaaagacattagacggtaagaagagatcgtgtaaggaccacaaggcccgaacagtagtgtctcccat
gtgattcgttgcacaagcaggcggtgtgcccggctagctcagtcggtagagcatgagactcttaatctcagggtcgtgggttcgagcccca
cgctgggcgaggtagaattttgtctccctgcagattcaagatgcctttgttgttatgaaatctacttaacggacgtagctgcatagcacgctgc
ctggctctactacagtctcagagaaacgatactggaagctgcaggtgattcggtaaacaacggtaaaatgccttgttctgaaagtacaggtg
tcgttcgttgttgagtacaccggatatgtgtgagacatgatggggagcagtaatgacgcttcttagttgcaaattttagtcgctaatacatgtt
attttcaggtgaagccagacgattcacatgcttgcatggacgaagtacccagaaggcacaacgggcgccgcctctttagctcagtggcaga
gcactggtctagtaaaccaggggtcgtgagttcgatcctcacaggaggcaaatgcattttggaaagcagttgcgcttcttggccatttcgaa
agttctttccagaaatacgaaaagttggcaatgggcatcaactacaatacaaatctcgcatgtgaatagggtggaatgttgttatttaagtag
gtcgcaaaacggcactgtttttggcggtcttaacgggatcgcgatgcctcttacttactcgtatcccttattttctacgagtaataaacactctcg
cgaatgtcgtcgtcgtcggcgccgccgccgcctcttgagcagtggtggtcaaacgccattgtatgacaagcagggagggaccacctgctgt
cgattgagaaggcagttagtgtttggtcctgatttcgaaactctgtttcatcagtcacatacgtggtggaaaatgacgggtgtataggaaaca
ggaagacgaagcgtcgcatttttagcagtgaattttgaggtcaagaatggcggcatagagggaaagcaccttcgttaaggtgtacgacgtt
tagaaatgagacgtatgtaaggaggtgttgaacgtcagtgaccgtgtggcctaatggataaggcgtcggacttcggatccgaagattgca
ggttcgaatcctgtcacggtcgtgtttttccacttctgcaaaagagacataccgttttactgtgatattagagcaatacaaaaccctctcaaagtt
tcagcgctccggctgcagtttggagaccagtttggagttgactgacaagac

>CSat16#Satellite/CSat16

atacagaagcattgtggtcactcgccaagctggggttaaatattttagcagaagatgtgaaacaagcaggagcctgtgtttcctttaggaga
gagcagtgtgtgctgtgcttttatcctttttgacgtttacttgggggctctgccatgtaatacaaagccttttgtagtaactgaatcacaggtt
ctcccctaacacttataaaaagaaacagatatccccc

>CSat17#Satellite/CSat17

gcagcctgggtagcctaagtggtaacaggaggataatactgttctcaaatttctgccaggtaggtgacacagggtgctacgccacagccta
aatattcgttatgtctctgtaatatatagggtgtggtcactcgccagtcatattgttaaaagagtaatttttaattaattaacgaaaatgaagctg
ggaagtatcattccaactcacctaccccaaaggagg

>CSat18#Satellite/CSat18

cggcgtccaatttcagacgtccataactaaataaatattcgtcgtcgcaatatcccactttcagcatccatagtacactacttatttagttatgtga
caaaaaatcaaatcatttctatggtcctattaggatctataggccactgaataccgaaggaaatacgcgggacgcctgggtgcgctct

>CSat19#Satellite/CSat19

aaatcgcaggtgaaatgcagaaaactcgactcaaatacaaattcagaaagtcgtccattatacacatttactatgtatgacaataaaacgtcg
tcgtcgtcgccgccgcacgcacgtcatatggaacacatgcggtaaaacacggaatagtgtctagaaacgcagggtgctaccgacagtatg
aaaattcgacagggtaccaactgaccaatactggacaggtgactcgatacaccgctgcattcgtactgtcctaggaggtctctaacgttgtct
gctgtgagtatatggacctgaaagtgaatactggttgtacattctgcactacatctttgcggcggcgtcttgcggacgtcaaacgagggcgg
agcttatggttgcgtagtgtggcgccagatacacatcaagaaacgagctacaagtatagtaaatacatgaaatgaggctaccacacgcaa
gtgtcaggatggccgagcggtctaaggcgccagactcaaggagcacccttggctgcaaatgcagcctgagccttctggtcctcgtctgag
ggcgtgggttcgaatcccactcctgacaaagattttactctttcgctacacaagtgtgcacatcgcagagaacgggtgtaatggggtaaaac
atgcagaaaccgcatcttgcgtggaacctacacctgccctatcaaactcaactctgcaatagtgtaagcttcaaacgcagagtactggttgca
aatgcggcgttaccctccgctgtctcactgacctccacgtcacagtcgcacaaatattataatgagcactcgagatcttacactgaatgttgtct
agacgcaaactgctaatgacaaatgcaaggaaacactacatccttaccactttacgaggttaaccctct

>CSat20#satellite/CSat20

gacccatgagcatgaatgacccaaatgacccatagaccatgacccataagacccaaaagacccaaaagacccaaactaaaagacccaaa
atgacccaaaagacccaaaagacccaaaagacctaaaagacccaaaagacccaaaagacccaaaagacccaaaagacccaagagccc
aaaagacccaaaaaacctaaaaaacacaaaaggcccaaaaaaaaccaaaaagaacaaaaagcccaaaaa

>5S_rDNA-1#rDNA/5S_rDNA-1

tactggtgtttccataagaaataacattaccgaaatgaacatgacattccatagtgatgaaaatacgagaaataatgggcgggcaaaagct
acgaaatgcactgtgagaagcacaaagctgtatgattccagaggcattttgtgaaataaagtagaacctgttactcagtagcagtgtaatttc
cccatttgtaaataaagtttatctcttttctcaccatgtccccaacgcttaatgccactgttttcgaacgtctcgccgcctctcgcagatgtttatcat
ccttttgctgtcatatgggccagaccccgagcggcagcgagcggcagcgagcaaagtcgggacaagtcgggacggaagggg aggggg
gcacctgcgaatgcagcttgcactcgctataaatgcctctaatatttgcgagttgcgtgtcgcgagtcttacctcgctagcagtggcgctctcc
agcacgacaatgcggtcgctgtgacgtgaagcatagtacgctgcaacgtcccgcctccctcgcggcaagtgactggcggtgatatagggc
agcccgcctcgcacgtggcggcccacctgccaattcggcagacgctcctggggcgcgaaaggcgcgcgccctgtggcgggagaagatg
cattccatcaaattaccgccctgtttctgccgaaaattccaactttaggtgtcaccagaggagcagaagccgattcctggcgcgccactacatt
ttactggcgccaacggccatacaacgttgaatacaccggttctcgtccgatcaccgaagttaagcaacattgggcccggttagtacttggat
gggtgaccgcctgggaaccgaaataaacacgaggtacaaaattgatgaaaatgggagaacaaatgagtgaagcaaaactaccaactgt
gctttgataataacgaagctgcatgagtgcagtgatattttctgaaatattacagagcccattactcagtagcagtgtaattttccgaatttgta
aataaaggtttatcttttatcgtaaaaaaatggtgctgttgagtctttttgtctcctatttcctactttctcccaccccccctccattgttaaactaccgt
tttcttgtgc

>5S_rDNA-2#rDNA/5S_rDNA-2

gtctgcgggcgctgttggcaagcggggtgcactcaacccttgtgaggcaaattcaggatcaacttgattgagaagtagcggctctggtctt
gaaaactgacatacggccgagagagcggtgtgctgaccacacaccactccataccgcagccggcgacgcatttgggccgaggatgacac
ggcggcctgtccgtaccgatgggcccttcgcggcctgttccgt

>CL170#Unclassified/CL170

tgttttcaaaataatccccagcaccgacacacagtatggtactcagtgtcagatacagcaggaaaatctgattgcagtgtagggcccaagtg
ttaaaaaaaattttagtatgcagcattgtagtacttgaacttcaaaaggaacaaacttcggaccctataaagtcaaaccaggaatcatatctga
cgcaataagtat
